# Targeted knockout of a conserved plant mitochondrial gene by genome editing

**DOI:** 10.1038/s41477-023-01538-2

**Published:** 2023-10-09

**Authors:** Joachim Forner, Dennis Kleinschmidt, Etienne H. Meyer, Jürgen Gremmels, Robert Morbitzer, Thomas Lahaye, Mark A. Schöttler, Ralph Bock

**Affiliations:** 1 Max-Planck-Institut für Molekulare Pflanzenphysiologie, Potsdam-Golm, Germany; 2 Institut für Pflanzenphysiologie, Martin-Luther-Universität Halle-Wittenberg, Halle (Saale), Germany; 3 ZMBP, Allgemeine Genetik, Universität Tübingen, Tübingen, Germany

**Keywords:** Genetic engineering, Transgenic plants, Plant breeding, Plant physiology

## Abstract

Fusion proteins derived from transcription activator-like effectors (TALEs) have emerged as genome editing tools for mitochondria. TALE nucleases (TALENs) have been applied to delete chimaeric reading frames and duplicated (redundant) genes but produced complex genomic rearrangements due to the absence of non-homologous end-joining. Here we report the targeted deletion of a conserved mitochondrial gene, *nad9*, encoding a subunit of respiratory complex I. By generating a large number of TALEN-mediated mitochondrial deletion lines, we isolated, in addition to mutants with rearranged genomes, homochondriomic mutants harbouring clean *nad9* deletions. Characterization of the knockout plants revealed impaired complex I biogenesis, male sterility and defects in leaf and flower development. We show that these defects can be restored by expressing a functional Nad9 protein from the nuclear genome, thus creating a synthetic cytoplasmic male sterility system. Our data (1) demonstrate the feasibility of using genome editing to study mitochondrial gene functions by reverse genetics, (2) highlight the role of complex I in plant development and (3) provide proof-of-concept for the construction of synthetic cytoplasmic male sterility systems for hybrid breeding by genome editing.

## Abstract (web summary)

By editing the mitochondrial genome and screening a large number of deletion mutants, this study shows that clean knockout mutants for a mitochondrial gene can be obtained and used to construct a synthetic cytoplasmic male sterility system.

## Main

Mitochondrial genome editing by direct genetic transformation is currently only feasible in very few species, with the unicellular green alga *Chlamydomonas reinhardtii* representing the only transformable photosynthetic organism^1,2^. The lack of tools to engineer plant mitochondrial genomes of seed plants has long hampered basic and applied research. While the availability of facile methods for chloroplast genome engineering has facilitated the in vivo analysis of all steps in plastid gene expression (for example, refs. ^3–5^), enabled functional genomics research by reverse genetics (for example, refs. ^6,7^), and facilitated new applications in biotechnology and synthetic biology (for example, refs. ^8–10^), similar studies in plant mitochondria have not been possible.

Recently, genome editing methods have been developed that, while not allowing transgene insertion into mitochondrial genomes, are capable of introducing mutations into mitochondrial genomes^11,12^. When targeted to mitochondria, transcription activator-like effector nucleases (TALENs) can cleave the mitochondrial DNA and thus induce deletions in the mitochondrial genome^13^. Base-editing methods relying on the fusion of TALEs with nucleoside deaminases^14,15^ have been shown to be capable of introducing specific point mutations into the mitochondrial DNA^16,17^. However, the isolation of genetically stable plants with a homogeneous population of mutated mitochondrial genomes (referred to as homochondriomic plants) has remained challenging^16,17^. In addition, mitochondrial base editors can cause substantial rates of off-target mutations in both the nuclear and the mitochondrial genomes^17,18^. Heritable homochondriomy of point mutations introduced into the plant mitochondrial DNA was recently achieved with a mutagenesis technique referred to as TALEN-gene-drive mutagenesis (TALEN-GDM)^19^.

For the study of gene functions by reverse genetics, genome editing reagents based on endonuclease fusions provide the most practical tools. However, their use in plant organellar genomes is less straightforward than in nuclear genomes due to the absence of a pathway for non-homologous end-joining (NHEJ)^13,20^. Thus, while appropriately designed TALENs can cleave mitochondrial sequences, the resulting double-strand breaks are repaired by homologous recombination^19^ or by microhomology-mediated recombination (MMR)^13^. The former restores the wild-type mitochondrial genome, while the latter has been shown to produce complex genomic rearrangements due to the involvement of distant repeats that cause large deletions encompassing essential mitochondrial genes. Stabilization of the edited genome, therefore, requires secondary genomic rearrangements to retain all essential genes that the initial deletion encompassed^13^. For these reasons, a clean knockout of a mitochondrial gene that maintains the wild-type conformation of the mitochondrial genome has not been achieved so far. Also, while the TALEN-based knockout of chimaeric cytoplasmic male sterility (CMS)-causing open reading frames and a duplicated (redundant) gene has been described^13,21^, the heritable homochondriomic knockout of a genuine mitochondrial gene function (that would cause an analysable mutant phenotype) has not yet been accomplished.

Here we report the targeted knockout of a conserved mitochondrial gene in tobacco (*Nicotiana tabacum*) plants. Using TALENs, we generated a large set of deletion mutants for the mitochondrial *nad9* gene encoding a subunit of complex I, the NADH-ubiquinone oxidoreductase complex in the inner mitochondrial membrane. Complex I is a major component of the respiratory chain. It provides a major entry point for electrons into the respiratory chain and is essential in many, but not all, organisms^22^. In tobacco, complex I is not critical for autotrophic growth, as evidenced by the isolation of several mutants lacking the complex^23,24^. While the severity of the plant phenotypes associated with loss of complex I varies between species^24–27^, plants in general possess NDH-2-type alternative NADH dehydrogenase that can replace complex I in terms of NADH oxidation and injection of electrons into the respiratory chain, albeit without pumping protons. Screening of our collection of *nad9* knockout mutants revealed lines that carried complex genomic rearrangements, but also clean deletions that retained the wild-type structure of the mitochondrial genome. We report the in-depth characterization of *nad9* knockout plants and their use to (1) assess the presence of promiscuous mitochondrial gene copies in the nuclear genome and (2) build a synthetic CMS system.

## Results

### TALEN-induced deletion of the mitochondrial *nad9* gene

We have recently reported the design of TALENs that target the mitochondrial *nad9* locus of tobacco and induce point mutations by TALEN-GDM^19^. The selection protocol developed for TALEN-GDM relied on the enrichment of point mutations by successive rounds of regeneration and the use of mutagenic agents. On the basis of previous reports on TALEN-induced deletions^13,21^, we reasoned that the *nad9* TALENs should also be suitable to pursue the isolation of *nad9* deletion mutants. We therefore selected a TALEN-expressing line (Nt-JF1006-30; containing a single TALEN arm targeting nucleotides 286–268 in the *nad9* coding sequence under the control of the CaMV 35S promoter^19^) and screened for *nad9* deletions by regenerating large numbers of shoots in tissue culture (see Methods and Supplementary Table 1 for details). In a subset of experiments (Supplementary Table 1), screening was conducted in the presence of rotenone, a specific inhibitor of complex I activity. The underlying assumption was that complex I inhibition by rotenone will cancel out the growth advantage that cells with wild-type mitochondrial genomes have over cells harbouring the *nad9* deletion (and that are probably complex I deficient).

**Fig. 1:**
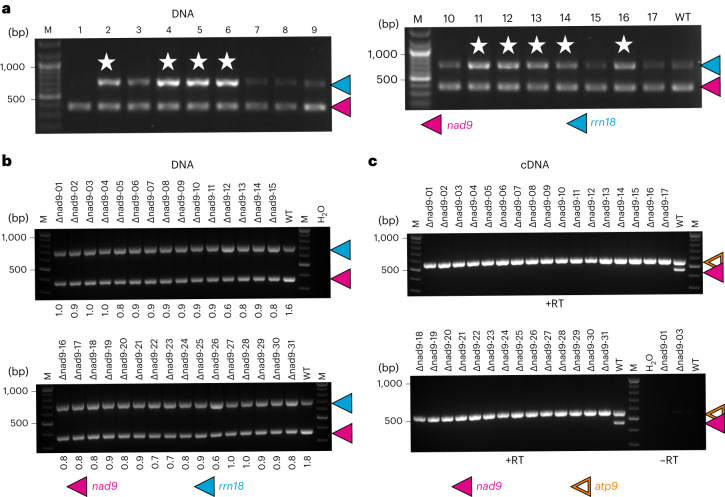
Isolation of tobacco mutants harbouring TALEN-induced deletions of the mitochondrial *nad9* locus. **a**, Targeted search for *nad9* deletion mutants. Due to the presence of NUMTs, even homochondriomic *nad9* deletion mutants will yield a *nad9* amplification product in genotyping PCRs. Therefore, semiquantitative ratiometric PCR was used to screen for deletion mutants using an *rrn18* amplicon as internal standard. In mitochondrial *nad9* deletion mutants, the relative PCR product intensities shift from dominance of the *nad9* amplicon in the wild type (WT) towards dominance of the *rrn18* amplicon in *nad9* deletion mutants (lanes indicated with star). Lanes 1–9 represent nine regenerated shoots subjected to primary screening for *nad9* deletions; lanes 10–17 are PCR assays of shoots derived from an additional regeneration round of a single identified deletion event (line Δnad9-01). M, DNA size marker. **b**, PCR genotyping of plants from all 31 identified Δnad9 lines (after backcrossing with the wild type as pollen donor). Numbers below the gel indicate the intensity of the *nad9* signal relative to the *rrn18* signal. H_2_O, buffer control. **c**, Expression analysis of plants from all 31 Δnad9 lines. *atp9* and *nad9* were co-amplified by RT–PCR in a single reaction. See text and Methods for details. The experiments in **a** and **b** were repeated independently at least once (with related but not identical plant material); the experiments in **c** were repeated once independently with similar results.

To identify *nad9* deletion lines, we developed a rapid screening method on the basis of semiquantitative ratiometric PCR. The rampant presence of *nad9*-containing mitochondrial DNA integrants in the nuclear genome (also referred to as promiscuous DNA or NUMTs: nuclear mitochondrial DNA)^19,28^ precludes the simple genotyping for presence/absence of *nad9* and instead necessitates the quantitative assessment of cellular *nad9* copy numbers. As *nad9* deletion from at least some copies of the mitochondrial genome is expected to decrease the ratio between (mitochondrial + nuclear) *nad9* copies and the copies of any other mitochondrial gene, we set up semiquantitative ratiometric PCRs with four primers per reaction. The two primer pairs co-amplify *nad9* and *rrn18*, the latter gene encoding the rRNA of the small subunit of the mitochondrial ribosome. Relative quantification of the two amplification products (that is, determination of the ratio of *rrn18:nad9* products) then identified lines in which the mitochondrial *nad9* had been deleted (Fig. 1a,b).

Screening of 288 regenerated shoots yielded 31 lines with clearly reduced relative *nad9* signal intensity (Fig. 1b). Seventeen of the lines were identified from screening of 268 regenerated shoots in experiments conducted in the absence of rotenone selection, and 14 lines were identified among 20 regenerated shoots in experiments performed under rotenone selection. Thus, rotenone selection appears to strongly increase the efficiency of mutant identification: while only 6.3% of the shoots carried a *nad9* deletion in the absence of rotenone selection, 70% of the shoots harboured a deletion upon rotenone selection.

The putative mitochondrial *nad9* deletion mutants (also referred to as Δnad9 lines) were subjected to additional rounds of regeneration to enrich the edited mitochondrial genome (which is resistant to TALEN cleavage) and select against the residual wild-type genome copies (which are cleaved and need to be repaired by homologous recombination with a wild-type genome or a mutated genome copy as template). Homochondriomy was assessed by semiquantitative ratiometric reverse transcription polymerase chain reaction (RT–PCR), which excludes the (transcriptionally silent) promiscuous *nad9* copies in the nuclear genome from amplification. For the ratiometric RT–PCR, the *atp9* complementary DNA (cDNA) was used as internal reference, which is less abundant than *rrn18* cDNA and therefore provides superior sensitivity (Fig. 1c). Homochondriomic *nad9* deletion lines could be isolated for all 31 lines, as evidenced by complete absence of the *nad9* amplification signal (Fig. 1c).

### Mitochondrial genome structure in *nad9* deletion lines

In the absence of an NHEJ pathway of DNA repair^13^, double-strand-break repair in mitochondrial genomes depends on homologous recombination (restoring the wild-type sequence) or microhomology-mediated end-joining. The nearly ubiquitous presence of microhomologies in the genome makes the structure of the mitochondrial DNA in the *nad9* deletion mutants largely unpredictable. To identify the breakpoints of the deletions, we initially selected three *nad9* deletion mutants (Δnad9-01, Δnad9-13 and Δnad9-29) for genome sequencing. As controls, the wild-type mitochondrial genome and the genomes of three independent allotopic complementation lines (Δnad9-c03, Δnad9-c10, Δnad9-c14, all derived from deletion line Δnad9-01) were also sequenced.

**Fig. 2:**
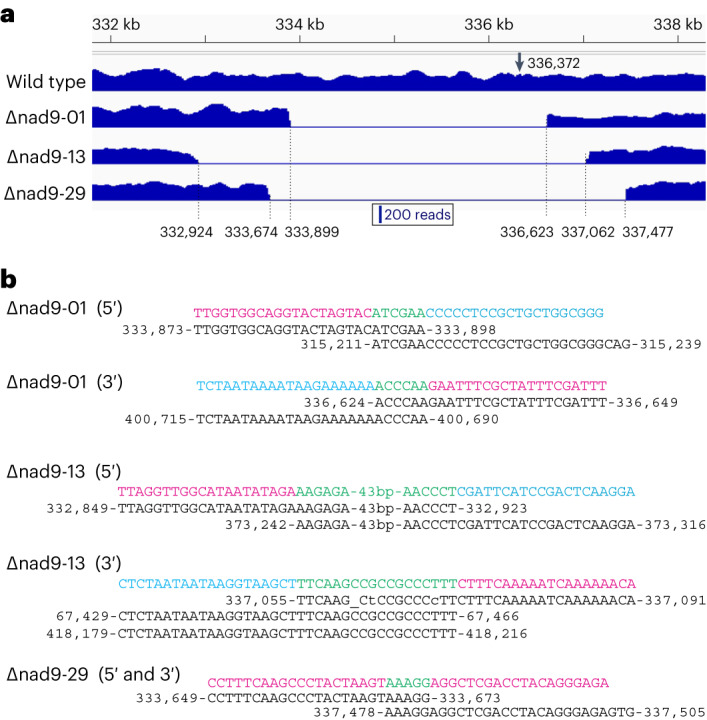
Mitochondrial genome conformations in three selected *nad9* deletion lines subjected to genome sequencing. **a**, Read coverage of the region surrounding the TALEN binding sites in the mitochondrial *nad9* gene. Nucleotide positions are according to the *N. tabacum* reference genome^64^ (NC_006581) and indicate the breakpoints of the deletions in the three mutant lines. The arrow marks the location of the TALEN cut site. **b**, Alignment of the sites of microhomology-mediated recombination involved in generating the *nad9* deletions in the three mutant lines. The breakpoint sequence in the *nad9* region is depicted in magenta; the distant sequence recombining with it is shown in cyan. For a graphical visualization of the recombination events in the genomes, see the physical maps in Extended Data Figs. 1–3. Note that in line Δnad9-29, no distant sequence is involved because the line represents a clean deletion that does not exhibit additional genomic rearrangements (Extended Data Fig. 3). The microhomologous sequences that probably triggered the recombination events are shown in green, mismatches are indicated by lowercase letters and insertions/deletions (InDels) by underscores.

**Extended Data Fig. 1:**
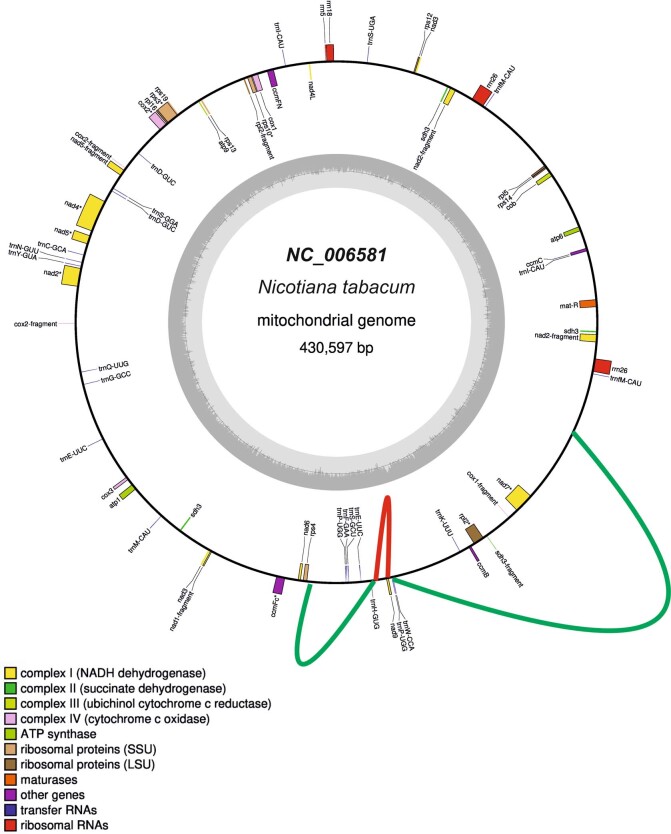
Mitochondrial genome configuration in mutant line Δnad9-01. Recombination events are marked in green and the net deletion is indicated in red.

**Extended Data Fig. 2:**
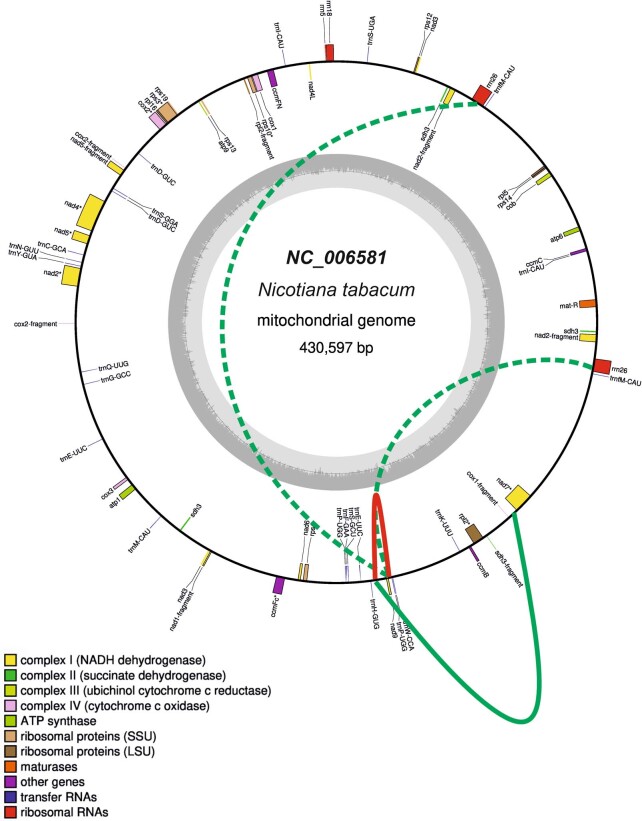
Mitochondrial genome configuration in mutant line Δnad9-13. Recombination events are marked in green and the net deletion is indicated in red.

To avoid artefacts from the abundant presence of NUMTs in the nuclear genomes, mitochondria were purified from all plant lines, and extracted mitochondrial DNA was used for genome sequencing. The three *nad9* deletion lines differed markedly in the upstream and downstream borders of the deleted region around the *nad9* gene (Fig. 2a and Extended Data Figs. 1–3), confirming that they represent independent TALEN-induced deletion events. Absence of any sequencing reads mapping to the deleted region ultimately confirmed that the lines are homochondriomic for the respective *nad9* deletions.

Inspection of the breakpoints of the deletions revealed the presence of microhomologies in all cases (Fig. 2b). In lines Δnad9-01 and Δnad9-13, recombination events with distant regions of the mitochondrial genome had occurred, leading to complex genomic rearrangements similar to those described in a previous study^13^. In both lines, the sequence upstream of *nad9* became joined to a distant region of the mitochondrial genome by microhomology-mediated recombination, and another MMR event joined the sequence downstream of *nad9* to another distant genomic region (Fig. 2b, and Extended Data Figs. 1 and 2). By contrast, genome sequencing of line Δnad9-29 revealed that this line represents a ‘clean’ *nad9* deletion in that the two MMR breakpoints reside immediately upstream and downstream of *nad9*. Consequently, the MMR event leading to *nad9* deletion does not comprise any other (essential) mitochondrial gene and, therefore, no additional recombination events are triggered (Fig. 2b and Extended Data Fig. 3). Thus, the conformation of the mitochondrial genome in line Δnad9-29 is wild-type-like, making this line particularly valuable in that it lacks any rearrangements that hamper the interpretation of the functional consequences of the *nad9* knockout.

### Phenotype of mitochondrial *nad9* deletion plants

**Fig. 3:**
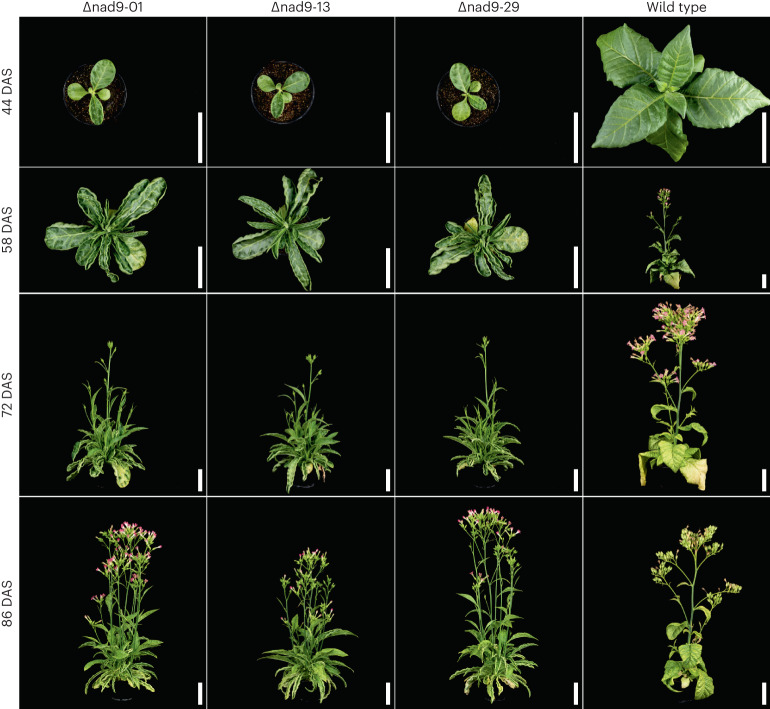
Phenotype of selected *nad9* deletion lines in comparison to the wild type. Plants were transferred to high-light conditions (1,000 µE m^−2^ s^−1^) 37 d after sowing (DAS) and grown in a controlled-environment chamber. Photographs were taken at the indicated time points. Scale bars, 10 cm.

**Extended Data Fig. 4:**
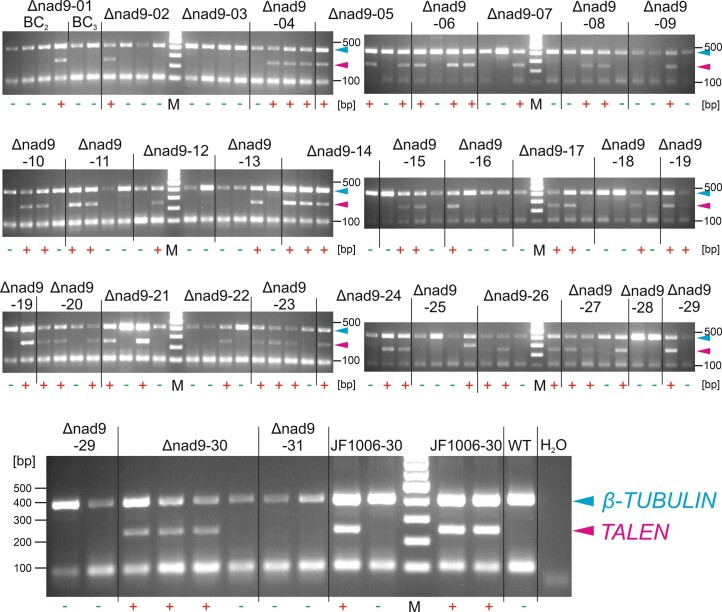
Identification of TALEN-free plants in the segregating progeny of crosses between mitochondrial *nad9* deletion mutants and the wild type (as pollen donor). Analyzed plants are from the second (BC_2_) or third backcross (BC_3_) with the wild type. Genotyping for presence of the TALEN transgene was performed by PCR using *β-TUBULIN* as internal control. Cyan arrowhead, *β-TUBULIN* PCR product (412 bp); magenta arrowhead, TALEN-specific PCR product (251 bp); M, DNA size marker; WT, wild type; H_2_O, buffer control; JF1006-30, TALEN control line harboring the wild-type mitochondrial *nad9* allele. In the strict sense, the experiments presented here were not repeated independently.

To evaluate the phenotype resulting from *nad9* deletion, the entire set of 31 mutants was first grown under standard greenhouse conditions. All 31 deletion lines displayed distinct macroscopic phenotypes (Supplementary Figs. 1–3). At the vegetative stage, growth and development were substantially retarded compared with the wild type, and leaves were more slender and showed rolled-up leaf margins (Supplementary Figs. 1–3). When grown under high-light conditions (see Methods) to trigger maximum growth rates, the mutant phenotype was even more severe and additional defects were observed, including pale leaf patches and reduced apical dominance (that is, increased branching; Fig. 3). Although these phenotypes were seen in all 31 deletion lines, the severity of the mutant traits was somewhat variable between lines (Supplementary Figs. 1–3). This observation may indicate that some lines harbour mitochondrial genome rearrangements that affect the severity of the mutant phenotype and/or could be indicative of variable penetrance of the mutant traits. Plant phenotypes (Supplementary Figs. 1–3) were assessed in the BC_2_ generation after two consecutive backcrosses of the initially recovered T_0_ mutant with wild-type plants (using the wild type as pollen donor). These backcrosses were conducted to (1) eliminate the TALEN construct from the nuclear genome and (2) largely exclude the possibility that tissue culture-induced mutations in the nuclear genome (somaclonal variation) are causally responsible for the observed phenotypic variation. The resulting TALEN-free plants (Extended Data Fig. 4) still showed substantial phenotypic variation between lines (Supplementary Figs. 1–3), preliminarily indicating maternal inheritance and suggesting a mitochondrial cause of the trait variation. Minor phenotypic variation was also observed within lines and even within individual plants, suggesting incomplete penetrance of some aspects of the mutant phenotype.

**Fig. 4:**
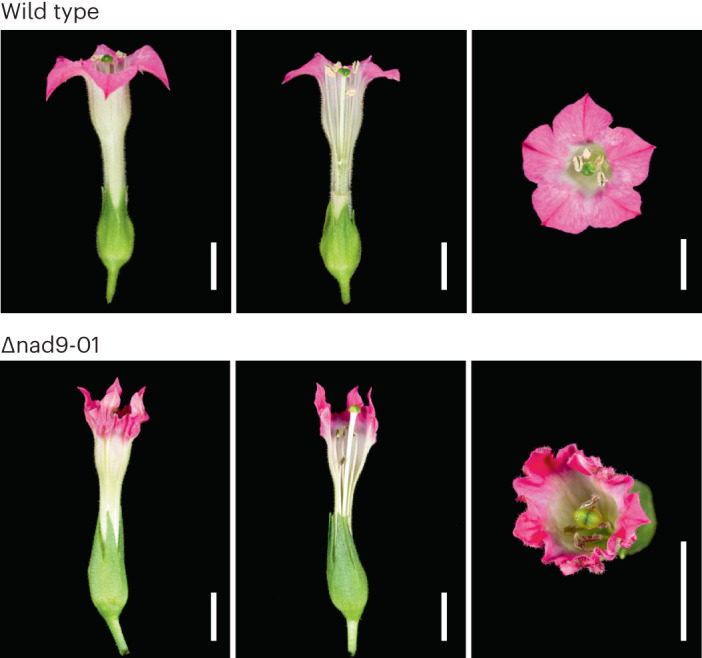
Typical flower phenotype of *nad9* knockout plants. Wild-type and mutant plants (*nad9* deletion line Δnad9-01) were grown under standard greenhouse conditions, and fully developed flowers were collected and photographed. Left: side view; middle: opened corolla; right: top view. Scale bars, 1 cm.

Particularly striking aberrations were seen in flower development. Petal shape was severely altered, the flowers showed curly corolla margins and vestigial anthers and the plants were male sterile (Fig. 4), indicating that the *nad9* deletion causes cytoplasmic male sterility (CMS).

Male sterility was occasionally slightly leaky in that a few seeds could sometimes be obtained. To quantify the level of sterility, pollen viability staining was performed with three independent homochondriomic *nad9* deletion lines. These assays revealed 0 viable pollen grains in line Δnad9-01 (out of 814 pollen grains stained) and line Δnad9-13 (out of 409 pollen grains) and 2 viable pollen grains in line Δnad9-29 (out of 1,026 pollen grains examined), confirming the very low male fertility of the *nad9* knockout plants. By contrast, all mutants were female fertile.

### Allotopic complementation of the *nad9* deletion

**Extended Data Fig. 5:**
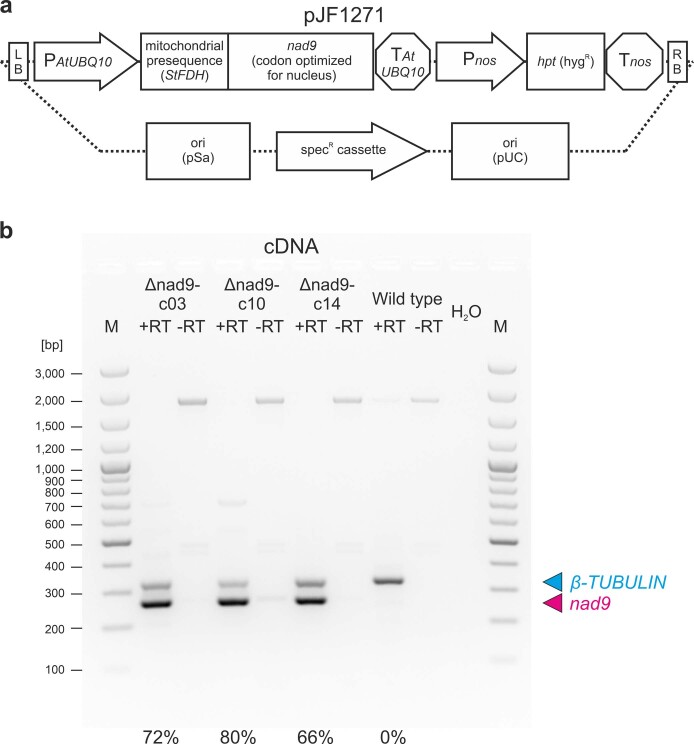
Allotopic expression of Nad9. (**a**) Structure of plant transformation vector pJF1271 used for allotopic complementation. LB, T-DNA left border; P_*AtUBQ10*_, promoter from the *Arabidopsis thaliana UBIQUITIN10* gene; *StFDH*, *Solanum tuberosum FORMATE DEHYDROGENASE*; T_*AtUBQ10*_, terminator from the *Arabidopsis UBIQUITIN10* gene; P_*nos*_, promoter from the *Agrobacterium tumefaciens* nopaline synthase-encoding gene; *hpt* (hyg^R^), gene encoding hygromycin phosphotransferase conferring resistance to hygromycin; T_*nos*_, terminator from the *Agrobacterium* gene encoding nopaline synthase; RB, T-DNA right border; ori (pSa) origin of replication in *A. tumefaciens*; spec^R^, spectinomycin resistance; ori (pUC) origin of replication in *E. coli*. (**b**) Analysis of the expression level of the nuclear *nad9* copy in the complemented lines by semiquantitative RT-PCR. *β-TUBULIN* expression served as internal control. The numbers at the bottom of the lanes indicate the relative intensity of the *nad9* signal (given as percentage of the total signal). M, DNA size marker; +RT, reverse-transcribed RNA; -RT, control reactions without addition of reverse transcriptase; H_2_O, buffer control; cyan arrowhead, *β-TUBULIN* RT-PCR product; magenta arrowhead, *nad9* RT-PCR product. In the strict sense, the experiments presented here were not repeated independently.

**Extended Data Fig. 6:**
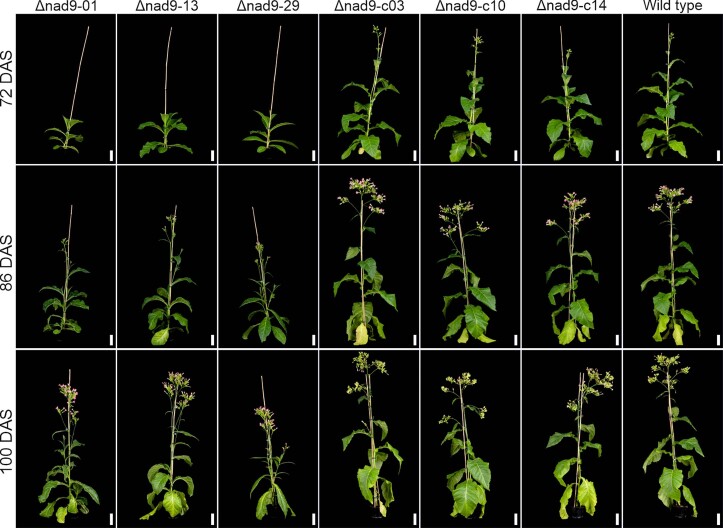
Growth phenotypes of three selected *nad9* deletion lines, three independently generated allotopically complemented lines and the wild type under standard greenhouse conditions. The images of wild-type and *nad9* deletion plants are from Supplementary Fig. 1. Plants were transferred to high light 37 days after sowing (DAS) and photographed at different time points. Scale bars: 10 cm.

To ultimately prove that the observed mutant phenotypes are caused by the absence of the Nad9 protein from mitochondria, allotopic complementation was attempted. To this end, a vector for allotopic expression of *nad9* from the nucleus under the control of the constitutive *AtUBQ10* promoter was constructed (Extended Data Fig. 5a) and introduced into a *nad9* deletion mutant (Δnad9-01) by biolistic transformation. Transgenic plants were selected and transgene expression was analysed by semiquantitative RT–PCR (Extended Data Fig. 5b). Three independently generated transgenic lines (Δnad9-c03, Δnad9-c10 and Δnad9-c14) that showed active expression of the nuclear *nad9* transgene were tested for rescue of the mutant phenotype. Both the growth phenotype (Extended Data Fig. 6 and Supplementary Fig. 4) and the flower phenotype (Supplementary Fig. 5) of the Δnad9-01 mitochondrial deletion line were fully rescued by the nuclear complementation construct, thus confirming that the observed phenotypes are causally linked to the loss of mitochondrial *nad9* gene function.

Crosses of the allotopic complementation lines with the wild type (using the wild-type plant as pollen donor) produced offspring that displayed the expected 1:1 Mendelian segregation for the *nad9* knockout phenotype. The nuclear transgene also fully restored male fertility. Thus, the mitochondrial *nad9* knockout in combination with the transgenically introduced *nad9* copy as nuclear restorer of fertility provides a synthetic CMS system^29–31^.

Next, we introduced the complementation construct in all other *nad9* deletion lines by pollination. In this way, we obtained complemented lines for all 31 mitochondrial *nad9* deletions. Importantly, due to the introduction by crosses, all 31 rescue lines harbour the identical nuclear *nad9* transgenic locus (in the identical genomic location) and therefore can be considered isogenic. Full rescue of the mutant phenotype was seen in 28 out of 31 mitochondrial mutant lines. The three remaining lines displayed varying degrees of growth retardation and incomplete rescue of the leaf phenotype (with leaves being more slender than in the wild type and one line still showing altered leaf margins). Also, one of the three partially rescued lines still exhibited aberrant flower morphology and another one was male sterile. These phenotypes did not segregate in the progeny of the crosses, consistent with maternal inheritance of the remaining genetic defects. These findings may suggest that the three incompletely rescued lines contain mitochondrial genome rearrangements that contribute to the severity of the mutant phenotypes.

**Extended Data Fig. 7:**
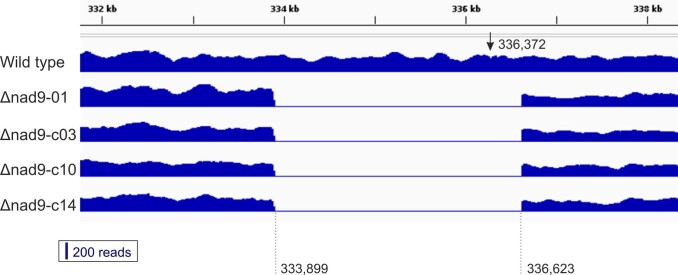
Mitochondrial genome conformations in the three allotopically complemented *nad9* deletion lines subjected to genome sequencing. The read coverage of the region surrounding the TALEN binding sites in the mitochondrial *nad9* gene is shown for the three complemented lines in comparison to the wild type and the *nad9* deletion line Δnad9-01 (cf. Fig. 2) used for allotopic complementation. The nucleotide positions of the breakpoints of the *nad9* deletion are given and refer to the *N. tabacum* mitochondrial genome sequence (^64^; NC_006581). The arrow marks the location of the TALEN cut site. The scale of the read numbers is indicated.

Sequencing of the three independent allotopic complementation lines revealed identical mitochondrial genome structures and sequences as in their progenitor line (*nad9* deletion line Δnad9-01; Extended Data Fig. 7). These data confirm that their restored wild-type-like phenotype was due to expression of the nuclear *nad9* transgene and furthermore suggest high stability of the engineered mitochondrial genomes.

### Biochemical characterization of *nad9* knockout plants

**Fig. 5:**
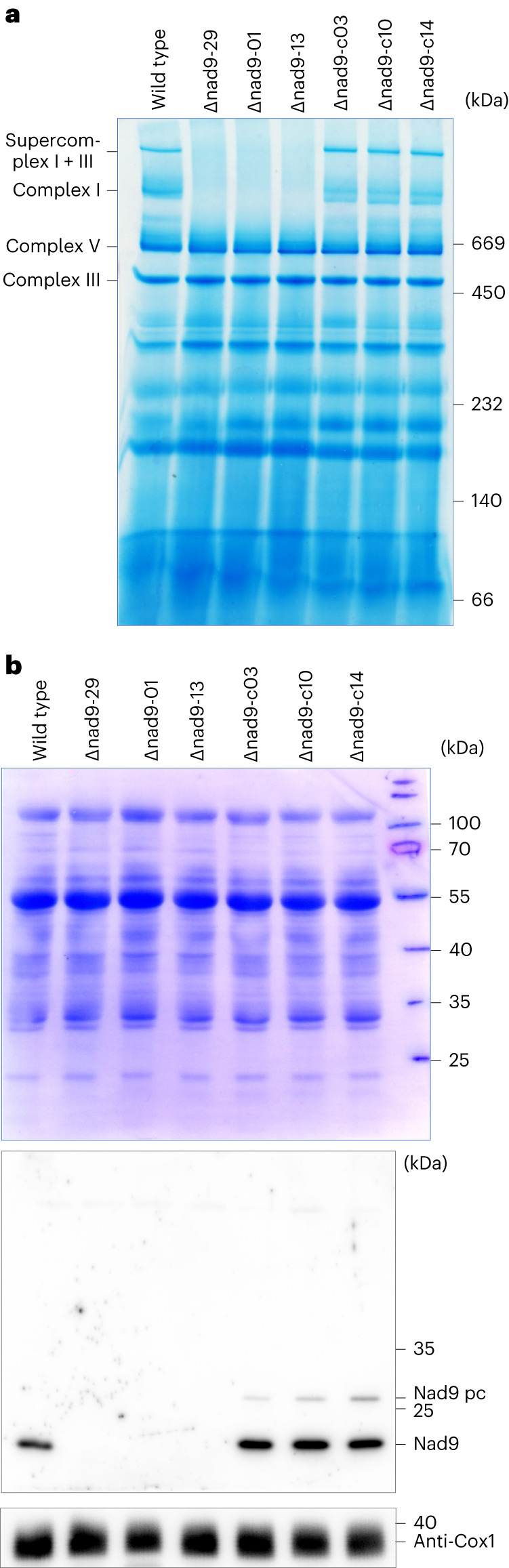
Analysis of mitochondrial protein complexes in three independent *nad9* knockout lines, three allotopically complemented lines and the wild type. **a**, BN–PAGE of mitochondrial membrane protein complexes. **b**, SDS–PAGE and immunoblot analysis of mitochondrial proteins. Electrophoretically separated proteins were stained with Coomassie (top) to confirm equal loading. Immunochemical detection of blotted proteins was conducted with anti-Nad9 antibodies (middle) and as a control for another respiratory protein complex, anti-Cox1 antibodies (bottom). Expected calculated protein sizes: 25.9 kDa for the unprocessed (transit peptide-containing) nucleus-encoded Nad9 precursor (Nad9 pc), 23.3 kDa for the processed Nad9, 22.8 kDa for the mitochondrially encoded Nad9 and 57.6 kDa (but approximately 40 kDa apparent size in SDS–PAGE) for Cox1. In total, the BN–PAGE was done three times, the Nad9 western blot two times and the Cox1 western blot once, with similar results (as technical replicates). Source data

To examine complex I accumulation in the absence of the mitochondrial *nad9* gene, we purified mitochondria and analysed mitochondrial protein complexes in three independent *nad9* deletion mutants, three independent allotopically complemented lines and the wild type by native gel electrophoresis and immunoblotting (Fig. 5). Blue-native polyacrylamide gel electrophoresis (BN–PAGE) analysis showed virtually complete absence of complex I from the three deletion mutants and full restoration in the complemented lines (Fig. 5a). Protein separation by sodium dodecyl sulfate–polyacrylamide gel electrophoresis (SDS–PAGE) followed by immunoblotting using an anti-Nad9 antibody revealed complete absence of the Nad9 protein from the deletion lines and full restoration to wild-type-like levels in the complemented lines (Fig. 5b). Interestingly, an additional weak Nad9 signal was detected in all three complemented lines. It has a slightly larger size than the mature Nad9 protein and most probably corresponds to the precursor protein from which the transit peptide sequence for protein import into the mitochondrial compartment has not (yet) been cleaved off.

Together, these data (1) further confirm the homochondriomy of our *nad9* deletion lines, (2) support an essential role of the Nad9 subunit for complex I accumulation and (3) indicate that none of the many promiscuous *nad9* sequences in the nuclear genome^19^ supplies functional Nad9 protein to mitochondria.

### Physiological characterization of *nad9* knockout plants

**Extended Data Fig. 8:**
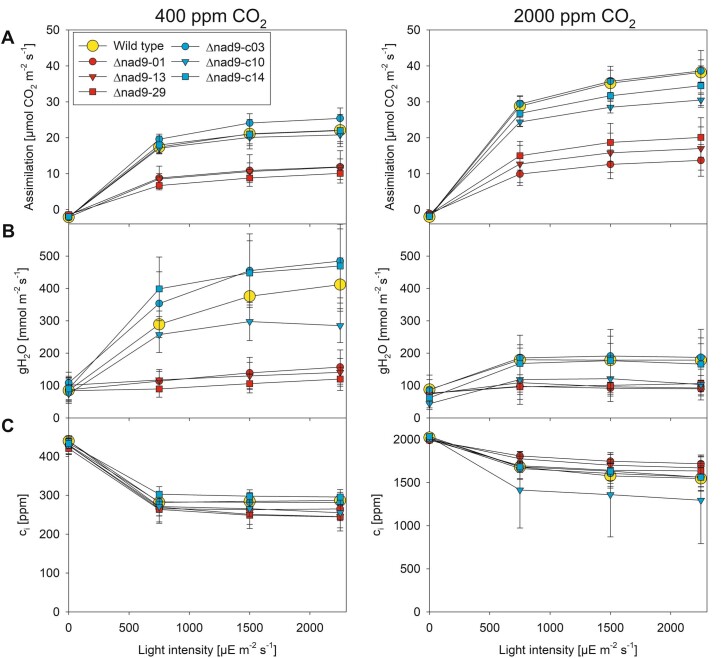
Characterization of gas exchange in mitochondrial *nad9* mutants in comparison to the wild type and three allotopically complemented lines. Measurements were performed at different light intensities and at ambient CO_2_ concentration (400 ppm) or at 2,000 ppm CO_2_, which normally fully suppresses photorespiration. However, it seems possible that the altered leaf anatomy in the *nad9* deletion lines (see text and Extended Data Fig. 9) leads to strongly increased mesophyll resistance to CO_2_ diffusion and precludes full repression of photorespiration in the mutants. (**a**) Assimilation rates. (**b**) gH_2_O, stomatal conductance. (**c**) c_i_, intercellular CO_2_ concentration in the substomatal cavity. Mean values and standard deviation are depicted. See Source Data files for a table with individual values and a version of this figure with individual data points shown. *n* = 6 biologically independent plants each examined over 1 experiment for 2,000 ppm CO_2_, *n* = 5 + 3 = 8 (WT), 6 + 1 = 7 (Δnad9-01), 5 + 1 = 6 (Δnad9-13), 5 + 1 = 6 (Δnad9-29), 5 + 3 = 8 (Δnad9-c03), 5 + 3 = 8 (Δnad9-c10), 5 + 3 = 8 (Δnad9-c14) biologically independent plants each examined over 2 independent experiments for 400 ppm CO_2_. Plants listed after the “+” sign (from experiment #2 for 400 ppm CO_2_) were used both for measurements at 2,000 ppm CO_2_ and 400 ppm CO_2_. Source data

Next, we wanted to assess the physiological consequences of complex I loss caused by deletion of the mitochondrial *nad9* gene. Previous analysis of the *Nicotiana sylvestris* CMSII mutant (lacking the Nad7 subunit)^32^ had revealed that, while photosynthetic defects at low growth light intensities were hardly detectable, adaptation to high-light conditions was severely impaired in complex I mutants, with the CMSII mutant showing strongly reduced assimilation capacity in high light^33^. This impairment in photosynthesis represents one of the most pronounced physiological consequences of the loss of complex I. We therefore decided to initially grow our set of three *nad9* deletion mutants, three allotopic complementation lines and the wild type under light-limited conditions and then shift them to high growth light intensities of 1,000 µE m^−2^ s^−1^. Photosynthetic parameters and gas exchange were measured before and after the shift to high-light conditions (see Methods, Supplementary Table 2 and Extended Data Fig. 8).

Upon growth at low light intensity (150 µE m^−2^ s^−1^), all measured photosynthetic parameters of the wild type, the *nad9* mutants and the complemented lines were very similar (Supplementary Table 2). Neither the chlorophyll *a*/*b* ratio of the mutants nor the maximum quantum efficiency of photosystem II (PSII) in the dark-adapted state (*F*_V_/*F*_M_) and the linear electron transport capacity (maximum rate of linear electron transport under light-saturated conditions) showed any consistent differences from the wild type and the three complemented lines. Minor differences in the *nad9* mutants, which, however, were at the borderline of statistical significance, were observed in the chlorophyll content per leaf area and the *P*_M_ parameter (a proxy of photosystem I (PSI)) contents per leaf area. Both values were slightly increased in the mutants (Supplementary Table 2).

After transfer to high light, plants were given a minimum of 10 d to acclimate and newly developed leaves were then analysed. Different from the reported characterization of the CMSII mutant^33^, both the *nad9* deletion mutants and the control plants (wild type and complemented lines) showed the typical adaptive responses to high light, including an increase in the chlorophyll *a*/*b* ratio and a strong increase in the capacity of linear electron transport. Remarkably, in the *nad9* mutants, chlorophyll content per leaf area, photosynthetic electron transport capacity and the *P*_M_ parameter were much more strongly increased than in the wild type and the complemented lines. However, the similar chlorophyll *a*/*b* ratios in the wild type, the *nad9* mutants and the complemented lines suggest that, except for the pronounced absolute increase in chlorophyll content in the *nad9* mutants, the relative composition of the photosynthetic apparatus is largely unaltered.

Surprisingly, even though photosynthetic electron transport capacity was elevated in the high-light-grown *nad9* deletion mutants, analysis of gas exchange parameters (Extended Data Fig. 8) revealed strongly reduced CO_2_ assimilation rates (at both 400 ppm and 2,000 ppm CO_2_) (Extended Data Fig. 8) and reduced stomatal conductance, while the intercellular CO_2_ concentration in the mutants was unaffected. As expected, no difference was seen between the wild type and the complemented lines (Extended Data Fig. 8).

### Altered leaf anatomy in *nad9* knockout plants

**Extended Data Fig. 9:**
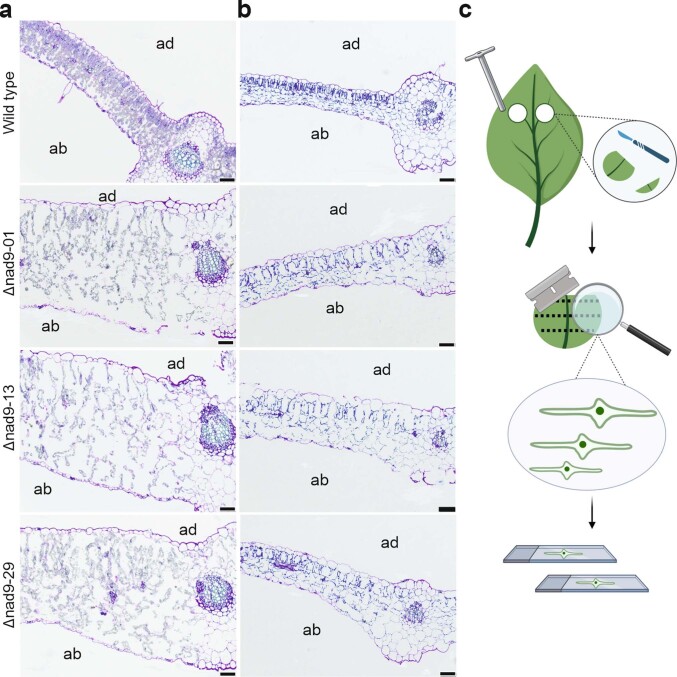
Altered leaf anatomy in *nad9* deletion lines. (**a**) Leaf cross sections from three *nad9* deletion lines and the wild type after transfer to high light. (**b**) Leaf cross sections from plants before transfer to high light (35 days after sowing). ad, adaxial side; ab, abaxial side; scale bars: 100 µm. (**c**) Illustration of the sample preparation procedure. Samples were taken from leaves of identical developmental stage and from the same area within the leaf. Created with BioRender.com. The experiments presented here in (a) and (b) were not repeated independently.

The striking discrepancy between increased photosynthetic electron transport capacity and reduced assimilation capacity in the high-light-grown *nad9* deletion mutants could be explained by a strongly increased resistance to CO_2_ diffusion within the leaf mesophyll. If this was the case, CO_2_ diffusion from the substomatal cavity (where the intercellular CO_2_ concentration is determined by gas exchange measurements) to the site of carboxylation would be restricted, as previously shown for the *N. sylvestris* CMSII mutant^34^. This could result from altered leaf anatomy, such as increased leaf thickness and/or reduced intercellular air space between mesophyll cells, both of which could also explain the substantially elevated chlorophyll content per leaf area in the *nad9* mutants. To investigate this possibility, leaf cross sections were prepared from three *nad9* deletion lines and the wild type both before and after the shift to high light (1,000 µE m^−2^ s^−1^) and studied by light microscopy (Extended Data Fig. 9). These analyses revealed striking alterations in leaf anatomy in the *nad9* knockout mutants. The palisade parenchyma is not properly developed, to the extent that palisade and spongy parenchyma look very similar (and are similarly disorganized). The vacuoles in the mesophyll cells of the mutants appear massively enlarged and the leaves are much thicker. The differences are seen both before and after the shift to high light, but they become more pronounced after the shift (Extended Data Fig. 9).

### Targeted identification of additional clean *nad9* deletions

**Extended Data Fig. 10:**
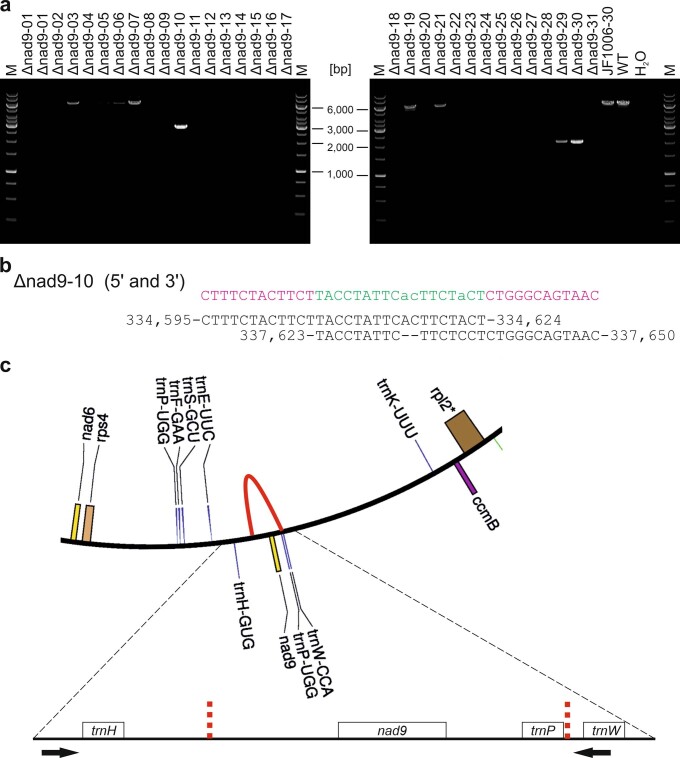
Targeted identification of *nad9* deletions by PCR and amplicon sequencing. (**a**) The region around *nad9* was amplified by long-range PCR with primers oJF1369 and oJF1374 (binding sites depicted in panel c; for sequences see Supplementary Table 4) from total genomic DNA of all *nad9* deletion lines (TALEN-free individuals; cf. Extended Data Fig. 4). Expected amplicon sizes are 5,742 bp for the wild-type mitochondrial DNA and 1,938 bp for the sequenced line Δnad9-29 (cf. Fig. 2). A PCR product smaller than the wild-type amplicon will appear only in those *nad9* deletion lines, where sequences upstream and downstream of *nad9* were directly fused by MMR (putative ‘clean’ deletions), but not in lines where the sequences upstream and downstream of *nad9* have undergone MMR with sequences elsewhere in the genome. Only lines Δnad9-10, Δnad9-29 and Δnad9-30 yield smaller PCR products. Note that the large 5,742 bp wild-type amplicon is not amplified in all lines, presumably because it is close to the upper size limit of standard PCRs. M, DNA size marker; WT, wild type control; H_2_O, buffer control (without DNA template); JF1006-30, TALEN control line (containing the wild-type *nad9* locus). (**b**) Alignment of the sites of microhomology-mediated recombination involved in generating the *nad9* deletion in line Δnad9-10. The breakpoint sequences in the *nad9* region are depicted in magenta, the microhomology is indicated in green. Sequencing of the Δnad9-10 PCR product revealed that the MMR event occurred in an imperfect 18 bp repeat (mismatches indicated as lowercase letters). (**c**) Deletion in the mitochondrial genome of mutant line Δnad9-10. The deletion of 3,014 bp (marked in red) encompasses the *nad9* locus and the downstream *trnP-UGG* gene (removing the genomic sequence between nucleotide positions 334,622 and 337,639). The linear physical map (not drawn to scale) shows the binding sites of the two primers (horizontal arrows) employed for PCR screening and the two sites that engaged in MMR (dotted vertical lines). See text for details. The experiments presented here in (a) were repeated twice independently with similar results (as technical replicates).

The isolation of line Δnad9-29 and the confirmation that it lacks any genomic rearrangement besides the *nad9* deletion (Extended Data Fig. 3) demonstrated that it is possible to generate clean mitochondrial knockout mutants whose phenotypes can be confidently attributed to the deletion of a specific mitochondrial gene. To determine whether the isolation of line Δnad9-29 was a fortuitous event, we screened all other *nad9* mutants for clean deletions using a PCR-based approach. To this end, PCR primers were derived from the two mitochondrial genes flanking *nad9* (*trnH-GUG* and *trnP-UGG* region; Extended Data Fig. 10) and used for amplification of the *nad9*-containing region. In this way, clean deletion events should be revealed by amplification of a product that is considerably smaller than the wild-type amplicon (5,742 bp). For line Δnad9-29, included as a positive control, the expected product size is 1,938 bp.

PCR screening of all 31 *nad9* deletion lines revealed small amplification products in three lines: Δnad9-29 (giving the expected band of 1,938 bp), Δnad9-30 (yielding a product of similar size) and Δnad9-10 (producing a band of ~3 kb; Extended Data Fig. 10a). DNA sequencing of the amplification products revealed the presence of identical deletions in Δnad9-29 and Δnad9-30, suggesting that the same MMR event occurred in these two lines. Line Δnad9-10 harbours a different deletion that probably also arose from MMR (Extended Data Fig. 10b). One breakpoint resides in the intergenic spacer between the transfer RNA (tRNA) gene *trnH* and *nad9*, and the other MMR event occurred in the spacer between *trnP* and *trnW* (Extended Data Fig. 10c). Thus, this deletion event encompasses the *trnP* gene, in addition to the *nad9* gene. Its homochondriomic state suggests that *trnP* is a non-essential mitochondrial gene. In fact, the tobacco mitochondrial genome contains two copies of the *trnP-UGG* gene: one copy resides in a tRNA gene cluster upstream of *nad9* and the other copy is located immediately downstream of *nad9* (Extended Data Fig. 10c). While the upstream copy represents the genuine mitochondrial *trnP-UGG* gene, the downstream copy is of chloroplast origin and was acquired by plastid-to-mitochondrial gene transfer^35,36^. The gene copy of chloroplast origin is deleted in line Δnad9-10 (Extended Data Fig. 10c). The deletion of this tRNA gene has no functional consequences in that the line shows the same mutant phenotype as lines Δnad9-29 and Δnad9-30, and its mutant phenotype is also fully complemented by expression of the nuclear *nad9* copy (see above).

### Systematic NUMT identification using the *nad9* deletion lines

The high copy number of the mitochondrial genome and the abundant presence of mitochondrial DNA sequences in the nuclear genome (NUMTs)^19,28,37^ often make it difficult to unambiguously identify the genomic location of amplified mitochondrial DNA sequences and also complicate the assessment of the complexity of nuclear integrants of mitochondrial DNA sequences. Having isolated homochondriomic *nad9* knockout plants that completely lack any mitochondrial *nad9* sequences, we reasoned that our mutants should provide a unique genetic material to determine the sequence complexity and copy number of *nad9* sequences that escaped to the nuclear genome of tobacco.

**Fig. 6:**
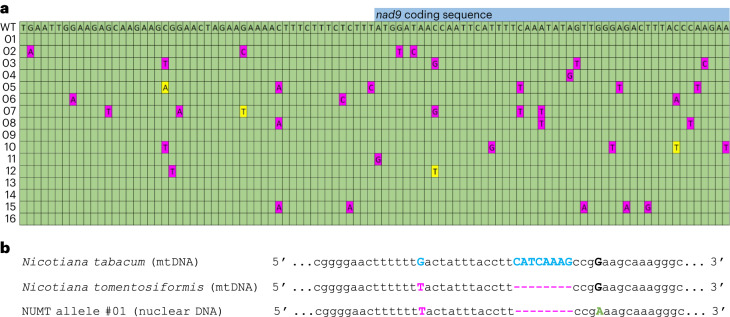
Abundant presence of promiscuous *nad9* copies in the nucleus (NUMTs). Taking advantage of the absence of a mitochondrial *nad9* locus in our *nad9* deletion mutants, nuclear *nad9* sequences could be selectively amplified from total DNA (of line Δnad9-29). The PCR product was cloned and 200 randomly picked bacterial clones were sequenced, yielding 16 different DNA sequences. While the consensus sequence of the NUMT alleles is identical to the mitochondrial *nad9* sequence, only one NUMT allele is identical to the mitochondrial *nad9* copy (thus probably originating from a recent transfer event). The sequence from positions 335,675 to 336,791 (in the reference mitochondrial genome; NC_006581.1) was analysed. The nucleotide positions of the *nad9* 5′UTR are 335,988–336,110, the positions of the *nad9* coding sequence are 336,111–336,683 and the positions of the *nad9* 3′UTR are 336,684–336,736. **a**, Partial alignment of the 16 identified NUMT sequences with the mitochondrial reference sequence (WT) around the *nad9* start codon. Nucleotide positions in the NUMTs that are identical to the mitochondrial *nad9* sequence are indicated by green cells, deviating positions are marked in magenta (with the mutated nucleotide indicated in the cell). When two different mutations are found at a given position, the second mutation is shown in yellow. For a full account of the sequence deviations from the mitochondrial *nad9*, see Supplementary Table 2. **b**, An NUMT predating the speciation event that produced *N. tabacum*. The mitochondrial *nad9* sequence of *N. tomentosiformis*, one of the diploid progenitor species of the allotetraploid species *N. tabacum*, was determined and aligned to that of the *nad9* locus of *N. tabacum* (whose mitochondrial DNA originates from the other diploid progenitor species, *N. sylvestris*). *N. tomentosiformis* exhibits two diagnostic sequence differences: the SNP G-335,722-T and the deletion ΔCATCAAAG (positions 335,735–335,742), marked in magenta. The corresponding *N. tabacum* nucleotides are marked in cyan. Of the 16 detected NUMT alleles, 1 shows the two *N. tomentosiformis* signature mutations, indicating that this mitochondrial sequence was transferred to the nucleus before the formation of *N. tabacum*. The sequence of the respective NUMT allele is shown. Note that it harbours an additional mutation (G-335,746-A; with the A marked in green, and the G marked in bold and black).

We therefore conducted PCRs with *nad9*-specific primers and total DNA extracted from *nad9* deletion lines as template, assuming that the amplified sequences will reflect the population of NUMTs in the nuclear genome. The amplification products were cloned (see Methods), and 200 bacterial colonies were randomly picked and sequenced. Of these, 174 harboured *nad9* sequences as inserts. Analysis of the 174 DNA sequences revealed 16 different alleles of *nad9* (Fig. 6a and Supplementary Table 3), suggesting an enormous complexity and a large number of *nad9*-containing mitochondrial DNA integrants in the tobacco nuclear genome. Interestingly, only 1 of the 16 alleles had a sequence identical to that of the mitochondrial *nad9* locus, indicating that the majority of gene transfer events are sufficiently old to have already accumulated some mutations after transfer to the nucleus (Fig. 6a and Supplementary Table 3).

In-depth bioinformatics analysis of the NUMT sequences revealed several additional interesting features. One of the sequenced alleles resembled the *Nicotiana tomentosiformis* allele of *nad9* (Fig. 6b). *N. tomentosiformis* is one of the diploid progenitor species of the allotetraploid species *N. tabacum*, but the two organellar genomes of *N. tabacum* (in the plastid and the mitochondrion) are from the other progenitor, *N. sylvestris*^38^. Thus, the presence of the NUMT representing the *N. tomentosiformis* mitochondrial *nad9* sequence suggests that this gene transfer event predates the speciation event that generated *N. tabacum* from *N. sylvestris* and *N. tomentosiformis*.

To obtain insights into the mechanism of mitochondrion-to-nucleus gene transfer of the *nad9* alleles and distinguish between DNA-mediated gene transfer and RNA/cDNA-mediated transfer^39–42^, we investigated the editing status of the 16 nuclear *nad9* sequences. To this end, we first determined the RNA editing pattern of the *N. tomentosiformis nad9* messenger RNA. All seven editing sites previously identified in the *nad9* coding region of *N. tabacum*^43^ were found to be conserved in *N. tomentosiformis*. When the editing status of the nuclear *nad9* alleles was assessed, all 16 sequences represented the unedited *nad9* sequence, strongly suggesting that their transfer to the nucleus did not involve an RNA or cDNA intermediate. A few isolated editing sites showed a T (seemingly reflecting the edited nucleotide), but in all of these rare cases, all other neighbouring editing sites in the same allele showed the unedited C, thus strongly suggesting that the isolated T represents a de novo mutation. This is unsurprising, given that C-to-T transitions are by far the most frequently occurring spontaneous mutations in plant nuclear genomes^44^.

## Discussion

Due to their independence of sequence specificity-conferring RNA molecules, TALEs currently represent the method of choice for the editing of mitochondrial genomes^11^. Our data reported here demonstrate that, by using TALENs targeted to mitochondria, it is possible to isolate clean knockout mutants of endogenous mitochondrial genes and obtain mutant phenotypes, whose analysis allows the characterization of mitochondrial gene functions. Previous work on TALEN-mediated genome editing in plants targeted non-conserved sequences: chimaeric CMS-causing reading frames and a duplicated (functionally redundant) gene^13,21,45,46^. By contrast, no heritable homochondriomic knockout of a genuine mitochondrial gene function has been obtained so far. Very recently, a TALEN-induced deletion line encompassing the *nad7* gene has been generated in *Arabidopsis*^47^. The single line described harbours a 5.5 kb deletion that, in addition to *nad7*, also removes two open reading frames of unknown function. Homochondriomy was reported to cause early lethality; hence no stable line could be established. The described lethality is in stark contrast to previous reports of complete complex I knockout mutants (of nucleus-encoded subunits) in *Arabidopsis* that have been shown to be viable and set seeds^25^, similar to our mitochondrial *nad9* deletion lines in tobacco. These discrepancies raise concerns about the large 5.5 kb deletion having caused additional molecular defects besides *nad7* knockout and loss of complex I. Moreover, the reported complex genomic rearrangements caused by microhomology-mediated repair of the double-strand breaks in the mitochondrial genome are a potential source of unintended effects and fitness penalties. Our observation that not all of our *nad9* deletion lines could be fully complemented by the nuclear *nad9* transgene adds to these concerns.

The generation of a large number of *nad9* deletion lines was key to the isolation of clean gene knockout events that (1) encompass only the gene of interest and (2) do not suffer from complicated genomic rearrangements. Clean gene deletions resulting from MMR events that involve sequences upstream and downstream of the *nad9* locus could be readily identified by screening the collection of deletion lines. In addition to two (identical) events of pure *nad9* deletion, we also obtained an event in which the deletion additionally encompassed the adjacent *trnP-UGG* gene. This gene originated from chloroplast-to-mitochondrial gene transfer and is redundant in that another *trnP* gene with the same anticodon is present in the genome and is obviously sufficient to serve mitochondrial translation. Whether the deleted *trnP-UGG* gene of chloroplast origin represents a pseudogene is currently unknown, but the full complementation of this deletion line by the nuclear *nad9* gene version suggests very strongly that the gene, even if functional, is dispensable.

The generation of a sufficiently large collection of *nad9* deletion lines was greatly facilitated by the selection conditions applied. Recovery of mitochondrial mutants was enhanced when selection was conducted in the presence of rotenone, a specific inhibitor of complex I. While *nad9* deletion mutants are unaffected by rotenone (due to their complex I deficiency; Fig. 5), the drug blocks complex I activity in wild-type cells and, in this way, prevents wild-type shoots from regenerating faster and overgrowing the mutant regenerants. This strategy should be considered in future studies that target other mitochondrial genes by reverse genetics. For example, on the basis of our findings reported here, we would expect that recovery of complex IV mutants will be greatly improved in the presence of cyanide, a specific inhibitor of cytochrome *c* oxidase.

The mitochondrial mutations isolated by our selection strategy attained the homochondriomic state very quickly, presumably due to the removal of the TALEN binding sites providing a strong replication advantage to the mutated mitochondrial genomes. Our findings that the three clean deletion events (lacking any secondary genomic rearrangements) were among the lines with the mildest growth phenotype (Supplementary Fig. 2) and could be fully complemented by introduction of the nuclear *nad9* transgene, underscores the importance of isolating such clean knockout events to obtain interpretable mutant phenotypes that can be clearly attributed to the function of the targeted mitochondrial gene.

It is also important to note that our approach described here allows the functional characterization of a set of neighbouring genes if (1) they are non-essential and (2) the collection of deletion events encompasses single and double deletions. For example, our recovery of a *nad9/trnP-UGG* double mutant, its comparative analysis with the *nad9* single mutant, and its allotopic complementation allowed us to also draw conclusions on the function of the *trnP-UGG* gene.

In addition to delayed vegetative growth, the *nad9* knockout plants displayed a range of developmental defects, including aberrant leaf shape, abnormal petal development and cytoplasmic male sterility. Some of these developmental aberrations were seen previously in natural CMS mutants^48,49^ and have been tentatively attributed to disturbed nuclear–mitochondrial interactions and retrograde signalling. The cytoplasmic male sterility of our *nad9* mutants and its rescue by expression of a nuclear *nad9* copy as restorer gene suggest that the introduction of suitable mitochondrial deletions with TALENs may become a useful approach to engineer CMS systems into crops where they are not naturally available. In view of the great importance of CMS to hybrid breeding^29,30,50^, this appears to be a worthwhile effort, with the availability of nuclear transformation being the only technical requirement that must be met.

Finally, we have shown that mitochondrial deletion mutants provide a unique genetic material to study the complexity of mitochondrial integrants in the nuclear genome and trace their evolutionary origin, age and fate. Our data revealed that at least one *nad9* NUMT in *N. tabacum* is older than the species and predates the allopolyploidization event that produced *N. tabacum* from *N. sylvestris* and *N. tomentosiformis*. Another NUMT appears to be very young in that its sequence is identical to that of the mitochondrial *nad9* locus (Fig. 6 and Supplementary Table 3). Overall, the large number of *nad9*-derived NUMTs confirms the high frequency of gene transfer from the mitochondrion to the nucleus. The 16 distinct NUMT alleles identified in this study are likely to even represent an underestimate because transferred partial *nad9* sequences were not captured by our experimental strategy, and some *nad9* NUMTs may have diverged so much from the mitochondrial *nad9* sequence (due to post-transfer accumulation of mutations in the nucleus) that they were excluded from PCR amplification and/or cloning. Another important finding that emerged from our analysis of promiscuous *nad9* sequences in the nucleus was that all of the 16 integrants result from direct DNA-mediated gene transfer, thus contrasting earlier reports on RNA/cDNA-mediated gene transfer from the mitochondrion to the nucleus^39,51^. While our data do not exclude the existence of RNA-mediated gene transfer, they suggest that DNA-mediated transfer is likely to be much more prevalent.

In summary, our work reported here demonstrates that clean deletion mutants for mitochondrial genes can be obtained by TALEN-mediated knockout and screening of a sufficiently large population of genome-edited lines for events that do not involve additional genome rearrangements. Our findings pave the way to systematic reverse genetic studies in plant mitochondrial genomes and also suggest a new strategy for building synthetic CMS systems in crops.

## Methods

### Plant material and growth conditions

*Nicotiana tabacum* cultivar Petit Havana was used for all experiments. The TALEN design and the TALEN-expressing line Nt-JF1006-30 were described previously^19^. For plant growth under sterile conditions, surface-sterilized seeds were germinated on Murashige and Skoog (MS) medium^52^ consisting of premixed MS salts and modified vitamins (Duchefa, M0245) and supplemented with 3% (w/v) sucrose (medium MS Suc3). The pH was adjusted to 5.8 and the medium was solidified with 0.68% (w/v) agar (Duchefa, M1002). For callus and shoot induction, the medium was additionally supplemented with 0.1 g l^−1^ 1-naphthylacetic acid and 1 mg l^−1^ 6-benzylaminopurine (medium NtSIM1). For rooting, regenerated shoots were transferred to MS Suc3 medium in Magenta boxes or, alternatively, to medium with 2% (w/v) sucrose (MS Suc2) supplemented with 1 mg l^−1^ indole-3-butyric acid. Plant growth in sterile culture was conducted in a diurnal cycle of 16 h at 25 °C and 25–50 µE m^−2^ s^−1^ light and 8 h at 20 °C in darkness, unless otherwise stated.

In the greenhouse, plants were grown on soil under standard conditions (16 h light, 8 h darkness, either at 25 °C day temperature, 20 °C night temperature and 55% relative humidity, or at 22 °C day temperature, 20 °C night temperature and 50% relative humidity; average light intensity: 300 µE m^−2^ s^−1^).

For physiological measurements, plants were cultivated for a minimum of 4 weeks after germination under long-day conditions (16 h light) at 22 °C and a light intensity of 150 µE m^−2^ s^−1^. At night, temperature was decreased to 18 °C, while the relative humidity was always set to 70%. After the plants had been transferred to pots of 13 cm diameter, they were moved to a high-light chamber (CONVIRON; 16 h light, 1,000 µE m^−2^ s^−1^, 22 °C, 75% relative humidity). During the night, the temperature was decreased to 18 °C and the relative humidity to 70%. Plants grown at 1,000 µE m^−2^ s^−1^ were also used for photographic documentation of phenotypes and for microscopic investigation of leaf cross sections.

### TALEN mutagenesis procedure

TALEN-induced deletion mutants for *nad9* were obtained by using a similar strategy as described for TALEN-GDM^19^. Briefly, leaf pieces from the TALEN-expressing line Nt-JF1006-30 (expressing a single TALEN arm targeted against nucleotides 286–268 in the *nad9* coding sequence under the control of the CaMV 35S promoter) were placed on shoot induction medium and individual regenerating shoots were genotyped (see below). Lines showing evidence of a *nad9* deletion were subjected to additional regeneration cycles until plants homochondriomic for the *nad9* deletion had been identified. In a subset of experiments, 40 µM rotenone was included in the regeneration medium to promote the recovery of complex I-deficient mutants (see text and Supplementary Table 1 for details). Some of the regeneration experiments were initially carried out to produce point mutants^19^ and therefore included treatment of leaf explants or seeds with ethidium bromide or *N*-nitroso-*N*-ethylurea (Supplementary Table 1).

### Identification of mitochondrial *nad9* deletion mutants

Pooled plant material harvested from three consecutive leaves per regenerated shoot was used to test for the presence of *nad9* deletions. Total DNA was extracted using the Extract-N-Amp extraction solution (E7526) and plant dilution solution (D5688, Sigma-Aldrich). First, the *nad9* locus was PCR amplified with primers oJF271 and oJF272 (Supplementary Table 4), and the PCR product (565 bp) was purified using the NucleoSpin Gel and PCR Clean-up kit (Macherey-Nagel) and sequenced (LGC Genomics) to exclude the presence of point mutations. Subsequently, plants were screened for a relative reduction in *nad9* by a semiquantitative PCR (with the same DNA sample as template) using a combination of four primers: oJF883 and oJF884 amplifying part of the mitochondrial 18S rRNA-encoding gene *rrn18* (yielding a product of 705 bp and serving as internal standard), and oJF273 and oJF499 (Supplementary Table 4) amplifying part of the *nad9* gene and giving a product of 372 bp. The PCR products were separated by electrophoresis in 1% agarose gels. Due to the presence of mitochondrial DNA sequences in the nucleus (see text for details), even plants with a complete deletion of the mitochondrial *nad9* gene produce the *nad9*-specific PCR product in this assay. Candidate plants carrying a deletion in the mitochondrial *nad9* locus were identified by a shift in the relative ratio of the *rrn18* and *nad9* PCR products (from dominance of the *nad9* amplicon in the wild type towards dominance of the *rrn18* amplicon in *nad9* deletion mutants). The shift in ratios was either determined by the naked eye or by quantifying the relative product intensities using Fiji (ImageJ 1.52i). DNA gel images were recorded with a Vilber Lourmat Quantum CX5 system, and contrast was adjusted in Adobe Photoshop CS5 Extended v.12.0.4 before quantification.

Semiquantitative PCR was also used to confirm the absence of *nad9* transcripts at the cDNA level. Material from three consecutive leaves was harvested from individual T_2_ plants, followed by extraction of total RNA using the NucleoSpin RNA plant kit (Macherey-Nagel) according to manufacturer instructions (but omitting the optional DNase digestion step). Reverse transcription was primed with oligonucleotides oJF1113 (binding to the *nad9* 3′UTR) and oJF1144 (hybridizing to the 3′UTR of the *atp9* locus used as internal control), and first-strand cDNA synthesis was performed with Super Script III reverse transcriptase (Invitrogen). The synthesized cDNA was purified with the NucleoSpin Gel and PCR Clean-up kit (Macherey-Nagel) and used as template for PCR with primers oJF496 (binding to the *nad9* CDS), oJF1145 (binding to the *atp9* 5′UTR) and oJF748 (an adapter-specific primer hybridizing to the 5′ terminal sequence of both oligonucleotides oJF1113 and oJF1144, thus excluding amplification of residual DNA present in the RNA preparations). Expected amplicon sizes were 568 bp for *atp9* and 465 bp for *nad9*. Products of the PCR reactions were separated by electrophoresis in 2% agarose gels. In this assay, mitochondrial *nad9* deletion mutants showed complete absence of the *nad9* RT–PCR product.

### Genotyping in the nucleus

Before nuclear supertransformation of line Δnad9-01, the recipient plants were tested for the presence of the TALEN transgene using oligonucleotides oJF401 and oJF481 (expected product size: 260 bp). In the same reaction, primers oAT02 and oAT09 (Supplementary Table 4) were included to amplify part of the endogenous *DHPS* gene (encoding dihydropteroate synthase) as internal control (expected amplicon size: 388 bp). PCR products were separated by electrophoresis in 1% agarose gels.

To test for the presence of the TALEN transgenes in second-generation backcrosses of the *nad9* mutant lines with the wild type (as pollen donor), PCR reactions with the TALEN-specific primers oJF1147 and oJF1323 (amplicon size: 251 bp) and a primer pair amplifying part of the endogenous *β-TUBULIN* locus (oligonucleotides oJF1028 and oJF1029; product size: 412 bp) as internal control were conducted, and products were analysed by electrophoresis in 2% agarose gels.

### Vector construction for allotopic expression of Nad9

A nuclear *nad9* complementation construct (plasmid pJF1271) was assembled using the GreenGate method^53^ from plasmids pJF1255, pJF1270, pGGE009, pJF1265 and pGGZ001. The *AtUBQ10* (At4g05320) promoter was amplified using primers oJF740 and oJF822 (Supplementary Table 4), the PCR product was cut with Eco31I and ligated into the similarly cut plasmid pGGA000, generating pJF1255. In contrast to pGGA006, the 3′-terminal guanine residue of the putative intron in *AtUBQ10* is present immediately upstream of the GoldenGate B-overhang (AACA) in pJF1255. A fusion gene consisting of the sequence encoding the 29-amino-acid N terminus of the *Solanum tuberosum* formate dehydrogenase (the first 25 of which represent the transit peptide that will be cleaved off after import of the protein into mitochondria^24^) and the complete *nad9* reading frame from the *N. tabacum* mitochondrial genome (cDNA sequence after RNA editing) was codon optimized for expression in the nucleus of *N. tabacum* and synthesized as a DNA string (GeneArt). The synthesized DNA was then amplified with primers oJF947 and oJF948 (Supplementary Table 4), digested with HindIII and EcoRI and ligated into the equally cut pGGA000 to create pJF1270. To generate pJF1265 (a GreenGate F-module with a P_*nos*_::*hpt*::T_*nos*_ hygromycin resistance cassette), the *hpt* coding sequence was amplified with primers oJF937 and oJF938. The backbone of pGGF007 was amplified with primers oJF939 and oJF940 (Supplementary Table 4), the two PCR products were digested with the restriction enzymes KpnI and SacI, and ligated. Plasmid pGGE009 contains the *AtUBQ10* (At4g05320) terminator as a GreenGate E-module^53^. The full sequence of pJF1271 has been deposited in GenBank under accession number OQ418153.

### Plant transformation

Nuclear transformation of line Δnad9-01 (after one backcross with the wild type as pollen donor, resulting in plants heterozygous for the TALEN transgene) was done by biolistics with a helium-driven particle gun (PDS-1000He, BioRad). Gold particles of 0.6 µm diameter were coated with 20 µg plasmid DNA (pJF1271), and 1,100 psi rupture discs were used for particle acceleration. Transformation events were selected on NtSIM1 medium (see above) supplemented with 30 mg l^−1^ hygromycin. To induce rooting, regenerated shoots were transferred to Magenta boxes with MS Suc3 medium containing 30 mg l^−1^ hygromycin. To eliminate possible chimaeric plants, an additional regeneration round in the presence of hygromycin was conducted. To this end, leaf explants taken from the primary transformant were placed onto NtSIM1 medium (supplemented with 30 mg l^−1^ hygromycin), and the resulting shoots were rooted in MS Suc3 with 30 mg l^−1^ hygromycin. All subsequent experiments were conducted with plants obtained in the additional regeneration round and their descendants. When hygromycin selection was applied during seed germination, the antibiotic was used at a concentration of 100 mg l^−1^. Fifteen independent transgenic events were selected, five of which were not self-fertile (with one of them showing a partial *nad9* knockout phenotype in leaves and flowers).

Relative expression levels of the nuclear *nad9* transgene were determined by semiquantitative RT–PCR using primers oJF1090 for the *nad9* transgene (product size: 284 bp), oJF1028 for *β-TUBULIN* (product size: 326 bp) and oJF748 as common reverse primer binding to the tags added by the 5′ extensions of oligonucleotides oJF1088 (*nad9*) and oJF1026 (*β-TUBULIN*) used for cDNA synthesis from extracted total RNA. PCR products were separated by electrophoresis in 2% agarose gels.

### Purification of mitochondria

Mitochondria were isolated from 40 g of fresh leaf material. To this end, the tobacco leaves were homogenized in extraction buffer (300 mM sucrose, 15 mM potassium pyrophosphate, 2 mM EDTA, 10 mM KH_2_PO_4_, 1% (w/v) PVP-40, 1% (w/v) BSA, 20 mM sodium ascorbate, 5 mM cysteine, pH 7.5) in a Waring blender, and the homogenate was filtered through two layers of Miracloth and centrifuged at 2,000 *g*. The supernatant was centrifuged again at 20,000 *g* and the pellet obtained was resuspended in wash buffer (300 mM sucrose, 1 mM EGTA, 10 mM MOPS-KOH, pH 7.2). After another centrifugation at 2,000 *g*, the collected supernatant was centrifuged at 20,000 *g* and the resulting pellet was resuspended in a small volume of wash buffer. The suspension was then loaded onto Percoll step gradients consisting of 1 volume of 50% (v/v) Percoll, 5 volumes of 25% (v/v) Percoll and 1 volume of 18% (v/v) Percoll (with all Percoll solutions prepared in gradient buffer composed of 300 mM sucrose and 10 mM MOPS-KOH, pH 7.2). The gradients were centrifuged at 40,000 *g* for 45 min and the mitochondria were collected from the interface between the 50% and 25% Percoll solutions. The mitochondria were then washed four times by resuspension in wash buffer, followed by pelleting by centrifugation at 20,000 *g*. The protein concentration of the isolated mitochondria was estimated using the Bradford method (ROTI-Quant, Carl Roth).

### Protein extraction and immunoblot analyses

Samples of 20 µg total mitochondrial protein were solubilized in loading buffer (80 mM Tris-HCl, pH 6.8, 25 mM EDTA, 0.1 M dithiothreitol, 1% (v/v) glycerol, 2% (w/v) SDS, 0.05% (w/v) bromophenol blue), electrophoretically separated in 12% SDS–PAA gels and then transferred to PVDF membranes (Immobilon-P, Merck Millipore) using a tank blotter (transfer buffer: 192 mM glycine, 25 mM Tris, pH 8.3). Transfer efficiency and equal loading were confirmed by Coomassie staining of the blotted membrane. Hybridization of the blots was performed with specific antibodies and chemiluminescence-based signal detection was conducted with secondary horseradish peroxidase-conjugated antibodies (ECL Prime, GE Healthcare). The Fusion-FX system (Vilber Lourmat) was used for image acquisition. Polyclonal antibodies against the complex I subunit Nad9 (ref. ^54^) were used at a dilution of 1:20,000, and polyclonal antibodies against the complex IV subunit Cox1 (ref. ^55^) were used at a dilution of 1:10,000. Anti-rabbit-HRP conjugate from Sigma (A0545) was employed as secondary antibody at a dilution of 1:10,000.

### Analysis of mitochondrial protein complexes by BN–PAGE

Mitochondrial membrane protein complexes were separated by BN–PAGE as follows: Purified mitochondria equivalent to 200 μg protein were solubilized with 5% (w/v) digitonin in solubilization buffer (150 mM potassium acetate, 10% (v/v) glycerol, 30 mM HEPES, pH 7.4) and loaded on a 4.5–16% gradient BN–PAA gel. Following electrophoretic separation, the protein complexes were stained with colloidal Coomassie, and the gel was scanned using a flatbed scanner.

### Gas exchange measurements

Gas exchange measurements were performed with a GFS-3000 gas exchange system equipped with the LED array unit 3056-FL as actinic light source (Heinz Walz). For the wild type and the complemented lines, the standard measuring head 3010-S with 8 cm^2^ leaf area was used. Because of the strongly lanceolate leaf shape of the complex I-deficient mutants (see Results), these leaves had to be measured in a custom-made cuvette originally designed for measurements with single *Arabidopsis* leaves (measuring area: 1 cm leaf width and 3 cm leaf length). While a flow rate of 750 µmol s^−1^ was used for the standard measuring head, the flow rate was reduced to 600 µmol s^−1^ for the custom-made cuvette.

Light response curves of CO_2_ assimilation were recorded at 22 °C cuvette temperature and 17,500 ppm humidity. Measurements were performed at two CO_2_ concentrations, 400 ppm and 2,000 ppm (with the latter usually being sufficient to fully repress photorespiration and determine the capacity of leaf assimilation). The youngest fully expanded leaves of plants grown for at least 10 d at 1,000 µE m^−2^ s^−1^ light intensity were used for the measurements. At this developmental stage, tobacco leaves have their highest capacity for both respiration and assimilation. Before measurements, plants were dark-adapted for 60 min to fully inactivate the Calvin–Benson–Bassham cycle. Leaf respiration was then determined in darkness for 30 min. Subsequently, the actinic light intensity was increased to 750, 1,500 and finally 2,250 µE m^−2^ s^−1^. Due to the long dark adaptation, at 750 µE m^−2^ s^−1^, leaves were measured for at least 75 min to reach the steady state of assimilation and stomatal conductance. After the final saturating illumination step, dark respiration was recorded again until a constant respiration rate had been reached.

### Analysis of photosynthetic electron transport

Parameters of photosynthetic electron transport were measured with the modular version of the Dual-PAM-100 instrument (Heinz Walz) at 22 °C. After 30 min of dark adaptation, the maximum quantum efficiency of PSII in the dark-adapted state (*F*_V_/F_M_) was determined. Then, light response curves of linear electron transport were measured via the chlorophyll *a* fluorescence. The measuring times at each actinic light intensity were 150 s under light-limited and 60 s under light-saturated conditions.

Linear electron transport was corrected for leaf absorptance, which was calculated as 100% incident light minus light transmitted through the leaf (%) minus light reflected on the leaf surface (%). Transmittance and reflectance spectra between 400 and 700 nm wavelength were recorded using an integrating sphere ISV-722 attached to a V-650 photometer (Jasco). The spectral bandwidth was set to 1 nm and the scanning speed was 200 nm min^−1^. Measurements of chlorophyll content and the chlorophyll *a*/*b* ratio were conducted with a Jasco V-630 photometer (Jasco) in 80% (v/v) acetone.

### Mitochondrial genome sequencing and bioinformatic analyses

For mitochondrial genome sequencing, DNA was isolated from aliquots of the mitochondrial preparations used for protein analyses using a rapid DNA mini preparation method^56^. Next-generation sequencing was carried out at the Sequencing Core Facility of the Max Planck Institute for Molecular Genetics, Berlin. After initial quality control using Bioanalyzer (Agilent), sequencing libraries were prepared from 12–90 ng of DNA per sample following the library preparation protocol of the KAPA DNA hyper prep kit (Roche) for double-indexed Illumina libraries. First, DNA was sheared using a Covaris S2 system (duty cycle 5%, intensity 5, 40 s run time). After end repair and A-tailing, Illumina sequencing-compatible adapters carrying unique dual indices were ligated to the fragments (NEXTFLEX Unique Dual Index Barcodes). Following bead-based clean-up steps, the libraries were amplified using seven cycles of PCR. After a second clean-up, library quality and size was checked with qBit, Bioanalyzer and qPCR. Sequencing was carried out in an Illumina MiSeq micro flow cell in PE150bp mode, yielding between 220,000 and 300,000 fragments per sample.

The initial quality check of the sequence data of all samples (in FASTQ format) was done with FastQC v.0.11.9 (https://www.bioinformatics.babraham.ac.uk/projects/fastqc/). Clipping of adapters was performed with CLCGenomicsWorkbench v.22 (https://digitalinsights.qiagen.com/products-overview/discovery-insights-portfolio/analysis-and-visualization/qiagen-clc-genomics-workbench/). The trimmed FASTQC files of all datasets were then mapped with bwa v.0.7.17 in mem mode (10.48550/arXiv.1303.3997) against the NCBI NC_006581.1 reference, followed by post-processing using SAMTools (v.1.14)^57^ (http://www.htslib.org/). Scanning for structural variants was done using DELLY (v.1.0.3)^58^ (https://github.com/dellytools/delly/) after marking duplicates with picard v.2.27.5 (https://github.com/broadinstitute/picard/releases/tag/2.27.5) and IGV (v.2.13.2)^59^ (https://software.broadinstitute.org/software/igv/) and by using the mpileup feature of SAMTools. De novo assembly of sample Δnad9-29 was done manually by removing the deleted sequence from the wild-type FASTA file (NC_006581.1). Annotation of the assembled sequences was conducted with the GeSeq tool (v.2.03)^60^, and OGDRAW v.1.3.1 was used for graphical representation of the genomes^61,62^ (https://chlorobox.mpimp-golm.mpg.de/).

### Leaf cross sections and microscopy

For the preparation of leaf cross sections, leaf discs of 5 mm diameter were taken from the youngest fully expanded leaves as illustrated in Extended Data Fig. 9c. Samples from seven plant lines (Δnad9-01, Δnad9-13, Δnad9-29, Δnad9-c03, Δnad9-c10, Δnad9-c14 and the wild type) were collected 35 d after sowing as timepoint zero before the shift to high light. For timepoint one, samples were taken 58 d after sowing (that is, after 21 d in high light) from the more quickly growing lines (Δnad9-c03, Δnad9-c10, Δnad9-c14 and wild type) and at 63 d after sowing (that is, after 26 d in high light) from the more slowly growing ones (Δnad9-01, Δnad9-13 and Δnad9-29) to obtain material from comparable developmental stages.

For fixation, the leaf explants were kept for ~2 h in a 5 ml reaction vessel containing 1 ml formalin-aceto-alcohol (FAA) solution (10% formaldehyde (37%), 5% acetic acid (100%), 50% ethanol (96%), 35% H_2_O). The four samples taken 58 d after sowing were kept for 5 d at 4 °C in the dark to be processed in parallel with the samples taken 63 d after sowing. Infiltration was done in a Leica ASP300S fully enclosed tissue processor and embedding in a Leica EG1160 tissue embedding station using Paraplast Plus. Cross sections were obtained by cutting the embedded samples in a Leica RM2265 automated microtome into slices of 4 µm thickness. Sections were transferred onto poly-l-lysine glass slides and dried on a heating plate at 42 °C overnight. Subsequently, the sections were stained with toluidine blue (steps: 10 min in Histo-Clear (I), 10 min in Histo-Clear (II), 1 min in 100% ethanol (EtOH), 30 s in 80% EtOH, 30 s in 60% EtOH, 30 s in 40% EtOH, 30 s in double-distilled water (ddH_2_O), followed by drying on a heating plate at 42 °C for 10 min; and staining with 0.05% toluidine blue by incubation at room temperature for 5 min, rinsing with ddH_2_O, drying on a heating plate at 42 °C for 10 min, 30 s in 60% EtOH, 30 s in 80% EtOH, 30 s in 100% EtOH, followed by drying on a heating plate at 42 °C for 5 min). Stained samples (two leaf pieces each) were analysed by light microscopy with an Olympus BX61 microscope.

### Assessment of pollen viability

Pollen viability in the putative CMS plants was determined according to previously published procedures^63^. Briefly, anthers from 2–10 mature flowers (depending on the amount of visible pollen) were collected from fully developed flowers and placed in a 5 ml reaction tube. Pollen was released by adding 1 ml of pollen viability solution (100 mM Na_3_PO_4_ and 1 mM Na_2_EDTA, pH 7.0) and vigorous vortexing. Larger tissue debris were manually removed using tweezers. The pollen was then pelleted by centrifugation for 5 min at 1,000 *g*, the supernatant was carefully removed and the pellet was resuspended in 100 µl staining solution (100 mM Na_3_PO_4_ and 1 mM Na_2_EDTA, pH 7.0, supplemented with 15 µM propidium iodide (PI, Abcam) and 30 µg ml^−1^ fluorescein diacetate (FDA, dissolved in acetone)). After 30 min incubation in the dark at room temperature, the pollen was collected by centrifugation (5 min at 1,000 *g*) and the supernatant was removed. The pollen-containing pellet was resuspended in 1 ml of pollen viability solution and the stained samples were then examined with a confocal laser-scanning microscope (Leica TCS SP8). For the FDA signal, a 488 nm argon laser was used for excitation, and emission was analysed at 500–550 nm. For PI, the dye was excited with a 561 nm argon laser, and emission was recorded at 600–650 nm. The signals from the individual channels, including the bright field, were recorded as separate images.

To count the number of viable cells, the intensity of the FDA and PI images was increased using Adobe Photoshop CS5 Extended v.12.0.4 by setting the input to 50 (out of 256) and leaving the output at 256. The modified images were then opened in Fiji (ImageJ 1.52i) and merged. Only pollen grains with a uniformly strong internal green signal were counted as viable.

### Identification of NUMT alleles

To determine sequences of nuclear mitochondrial DNA (NUMTs) that represent promiscuous copies of *nad9* in the nuclear genome, the *nad9* locus was amplified from total DNA with primers oJF469 and oJF470 (Supplementary Table 4) using the highly accurate Phusion high-fidelity DNA polymerase in buffer HF (Thermo Fisher). The PCR products were digested with BamHI and KpnI and ligated into the equally cut plasmid vector pGGA000 (ref. ^53^). Plasmid DNA was isolated from individual transformed *Escherichia coli* colonies and the insert sequences were determined by Sanger sequencing. In the first iteration, 38 different *nad9* NUMT inserts were obtained from 43 clones. We noticed that many of these inserts were chimaeric in that they shared different blocks of SNPs, suggesting that they were derived from in vitro recombination events. To reduce the risk of template switching (by incomplete extension of a primer molecule in cycle *n* −1 and reannealing of the partially elongated DNA strand to a different template molecule in cycle *n*), the extension time was increased from 20 s to 35 s in the second iteration. To reduce heteroduplex formation (when, in the last cycle of the PCR, two single-stranded product molecules anneal to one another instead of hybridizing to a primer molecule), the primer concentration was raised from 0.5 µM to 1 µM and the cycle number was reduced to 20 or 25. In addition, the annealing temperature was increased from 48 °C to 58 °C. Furthermore, PCR products were treated with T7 endonuclease I (NEB) before BamHI/KpnI digestion to specifically eliminate heteroduplexes. In iteration one, total DNA isolated from line Δnad9-01 was used as template and in iteration two, total DNA from line Δnad9-29 was used. As an internal control in four of the eight parallel reactions of iteration two, total DNA from the wild type was spiked in as template, so that inserts yielding the pure mitochondrial sequence would indicate the absence of in vitro recombination. In these control reactions, 57 inserts displaying the wild-type mitochondrial sequence were found, whereas only 4 such inserts were obtained from the parallel reactions where no wild-type DNA had been spiked in (which represent a recently transferred NUMT allele; Supplementary Table 3), indicating that in vitro recombination of different amplification products had been effectively prevented by the above-described countermeasures. The results from iteration two are reported in Fig. 6 and Supplementary Table 3. An insert was considered a bona fide NUMT allele when its sequence was found in at least three independent clones. Sequencing reactions were conducted with primers oJF060 and oJF061 (Supplementary Table 4).

The mitochondrial *nad9* sequence from *N. tomentosiformis* was determined by PCR amplification of the respective region with primers oJF469 and oJF470 (resulting in an amplicon of 1,176 bp), with total DNA as template, followed by Sanger sequencing. The cDNA sequence was obtained by reverse transcription of total RNA (isolated with the NucleoSpin RNA plant kit; Macherey-Nagel) primed with oJF1113 (Supplementary Table 4), followed by PCR amplification with oligonucleotides oJF311 and oJF748 (product size: 769 bp), and DNA sequencing.

### DNA sequence analyses and artwork

DNA sequence analyses were performed using the tools of the Lasergene suite (DNASTAR) v.14 and 17, SnapGene Viewer v.7.0.1 (https://www.snapgene.com/snapgene-viewer/) and BLAST (https://blast.ncbi.nlm.nih.gov). Extended Data Fig. 9c was generated with pre-drawn icons from BioRender.com (https://biorender.com).

### Reporting summary

Further information on research design is available in the Nature Portfolio Reporting Summary linked to this article.

### Supplementary information

Supplementary Information Supplementary Figs. 1–5 and Tables 1–4.

Reporting Summary

### Source data

Source Data Fig. 5 Unprocessed western blot for Fig. 5b (lowest panel).

Source Data Extended Data Fig. 8 Version of Extended Data Fig. 8 with individual data points.

Source Data Extended Data Fig. 8 Statistical source data (table listing the measured values on which Extended Data Fig. 8 is based).

## Figures and Tables

**Extended Data Fig. Fig. 3:**
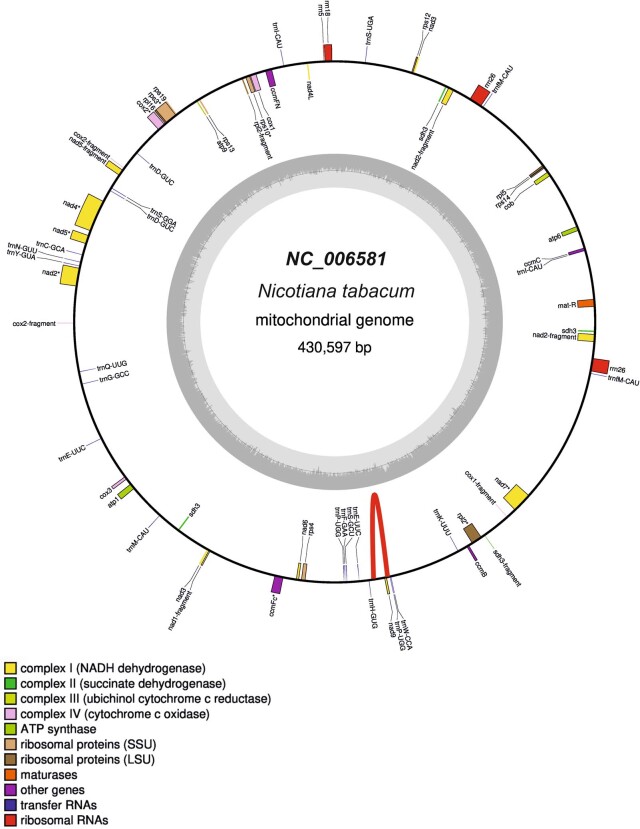
Mitochondrial genome configuration in mutant line Δnad9-29. The net deletion is marked in red.

## Data Availability

The data supporting the findings of this study are available within the paper and its supplementary information files. The full sequence of pJF1271 has been deposited in GenBank under accession number OQ418153. The NGS sequencing results are available at https://www.ncbi.nlm.nih.gov/bioproject/?term=PRJNA934725 as stated in the manuscript. Access is not restricted. NCBI entry NC_006581.1 was used as the mitochondrial reference genome for tobacco. Source data are provided with this paper.
