# Supplementary material for: Targeted knockout of a conserved plant mitochondrial gene by genome editing
Source: Nat Plants. 2023 Oct 9;9(11):1818–31. doi: 10.1038/s41477-023-01538-2 (PMC10654050; doi:10.1038/s41477-023-01538-2)
Supplement: Supplementary file 1 — Supplementary Figs. 1–5 and Tables 1–4. [file 41477_2023_1538_MOESM1_ESM.pdf]

nature plants

Article

https://doi.org/10.1038/s41477-023-01538-2

Targeted knockout of a conserved plant mitochondrial gene by genome editing

In the format provided by the authors and unedited

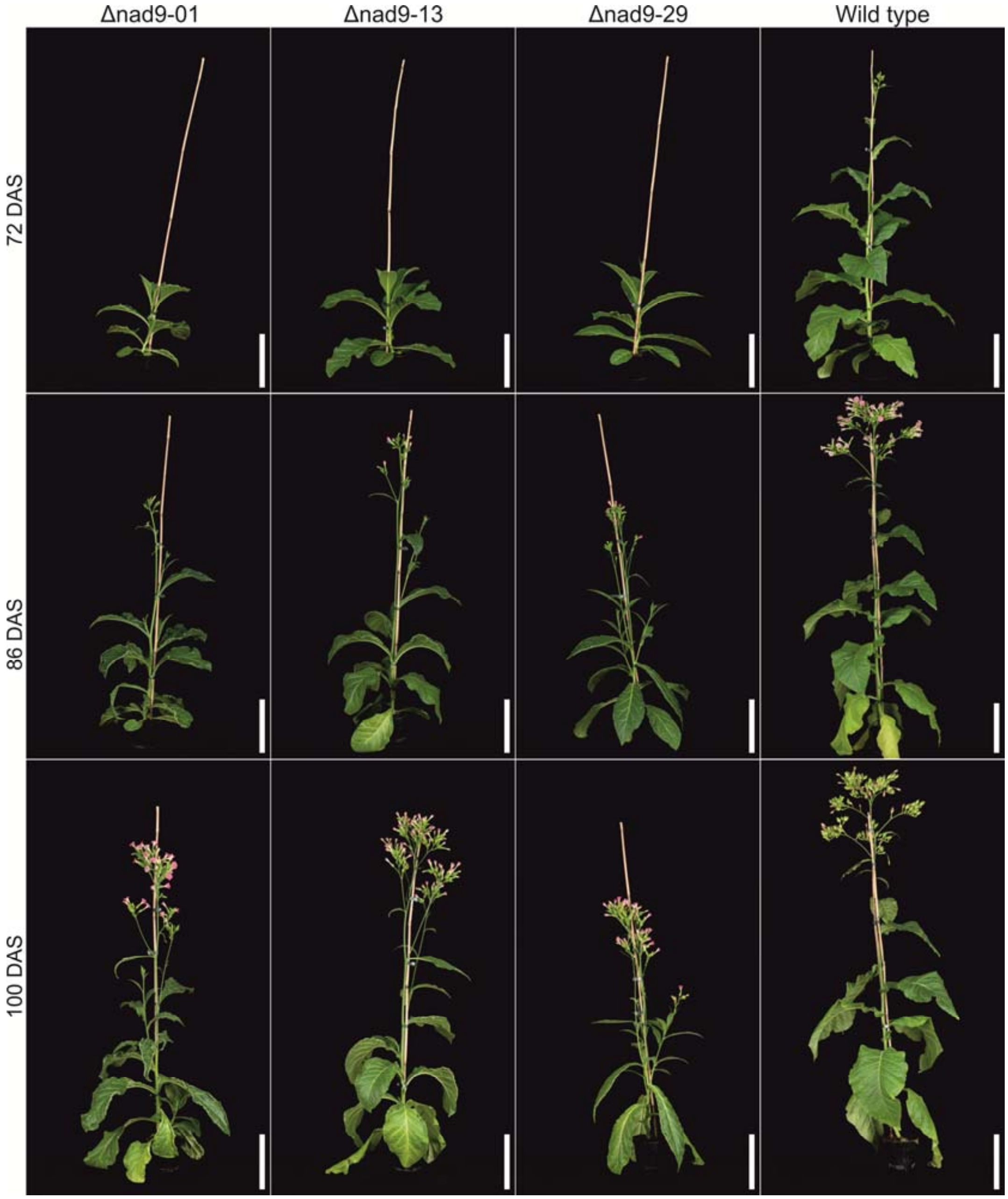

Supplementary Figure 1. Phenotype of selected *nad9* deletion lines (BC_2 generation) in comparison to the wild type. Plants were grown under standard greenhouse conditions (average light intensity: 300 µE m^{-2} s^{-1}) and photographed at different time points. DAS, days after sowing; scale bars: 20 cm.

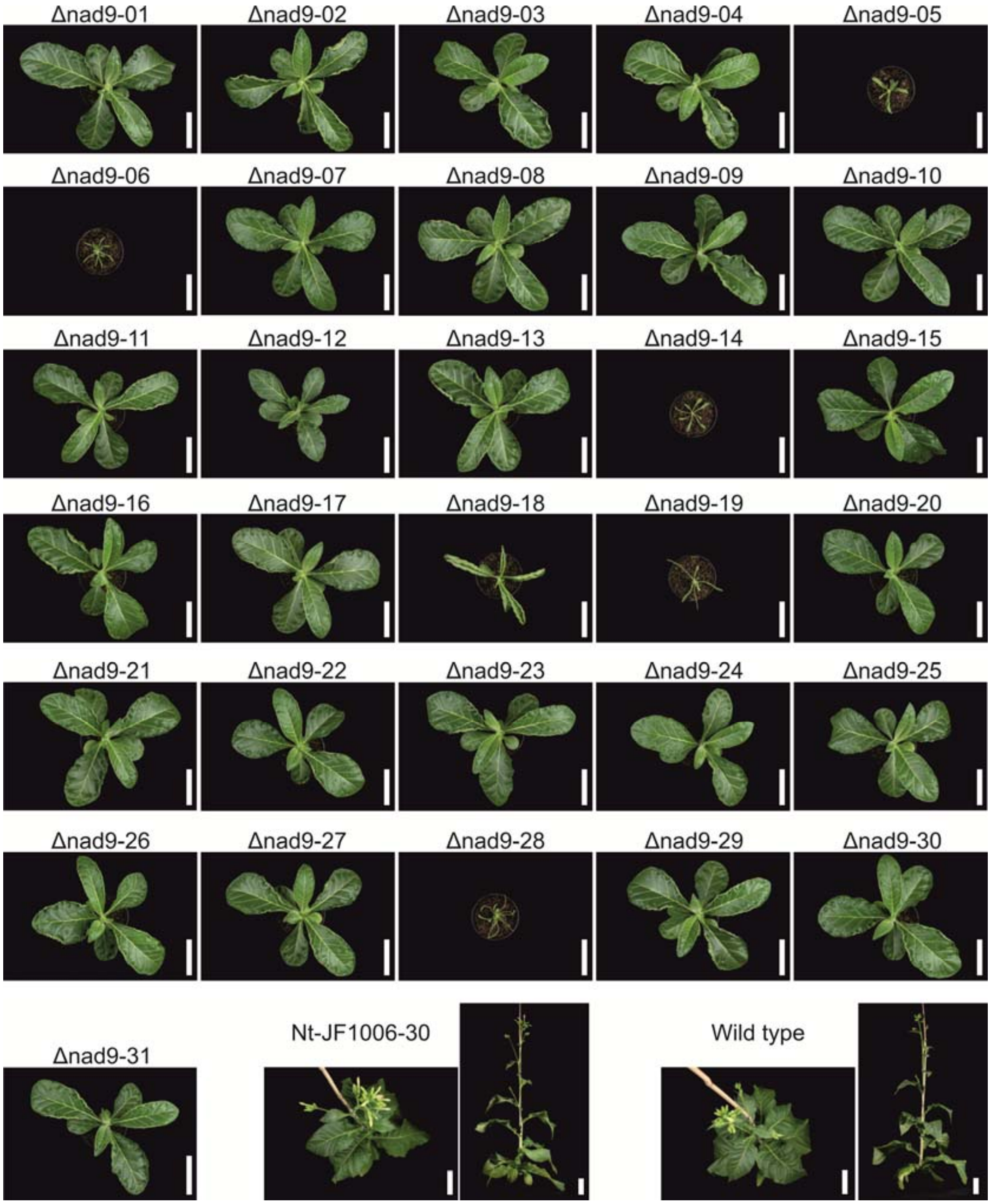

Supplementary Figure 2. Phenotype of all 31 *nad9* deletion lines (BC_2 generation), the control line Nt-JF1006-30 (harboring the wild-type *nad9*) and the wild type under standard greenhouse conditions. All *nad9* mutants shown are TALEN-free plants (i.e., derived from crosses that removed the TALEN construct from the nuclear genome). Plants were photographed 55 days after sowing. An additional side-view image is shown for the wild type and the TALEN control line, since these two lines grow significantly faster than the *nad9* knock-out mutants. Scale bars: 10 cm.

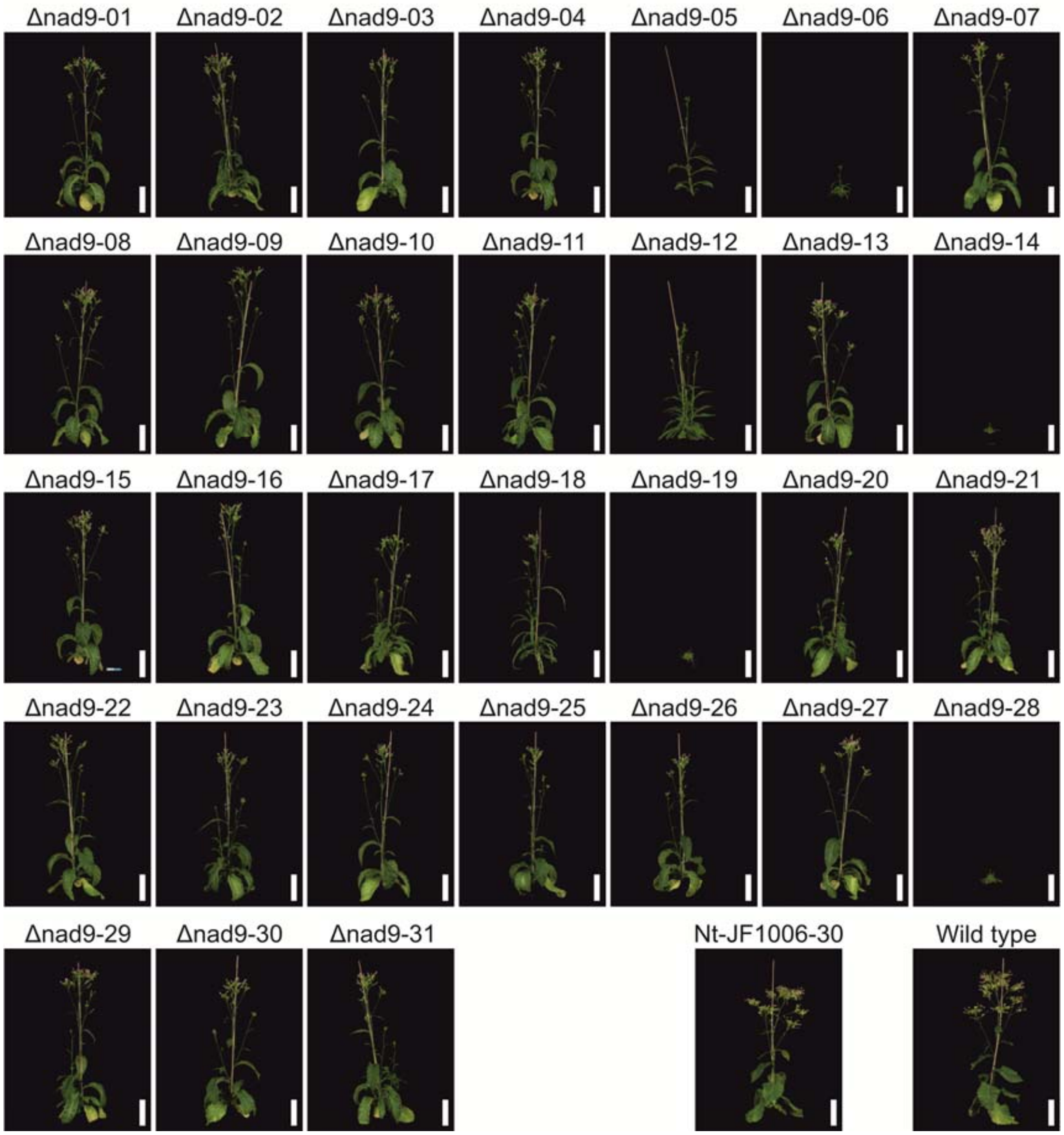

Supplementary Figure 3. Phenotype of all 31 *nad9* deletion lines (BC_2 generation), the TALEN control line (Nt-JF1006-30) and the wild type under standard greenhouse conditions. All *nad9* mutants are TALEN-free. Plants were photographed 83 days after sowing. See Supplementary Fig. 2 for further details. Scale bars: 20 cm.

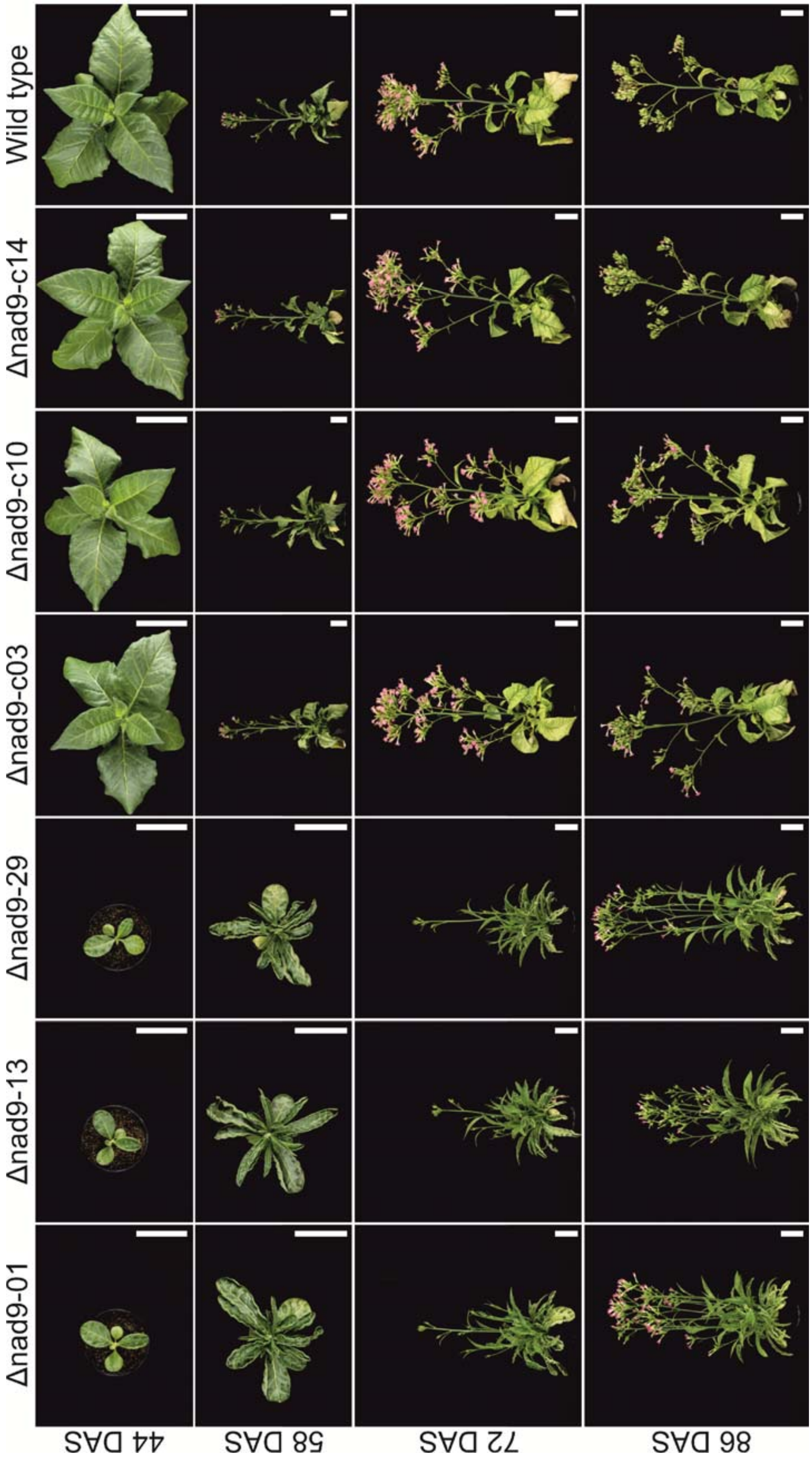
Wild type
Δnad9-c14
Δnad9-c10
Δnad9-c03
Δnad9-29
Δnad9-13
Δnad9-01
44 DAS
58 DAS
72 DAS
86 DAS

Supplementary Figure 4. Growth phenotypes of three selected *nad9* deletion lines, three independently generated allotopically complemented lines and the wild type under high-light conditions. The images of wild-type and *nad9* deletion plants are from Fig. 3. Plants were transferred to high light 37 days after sowing (DAS) and photographed at different time points. Scale bars: 10 cm.

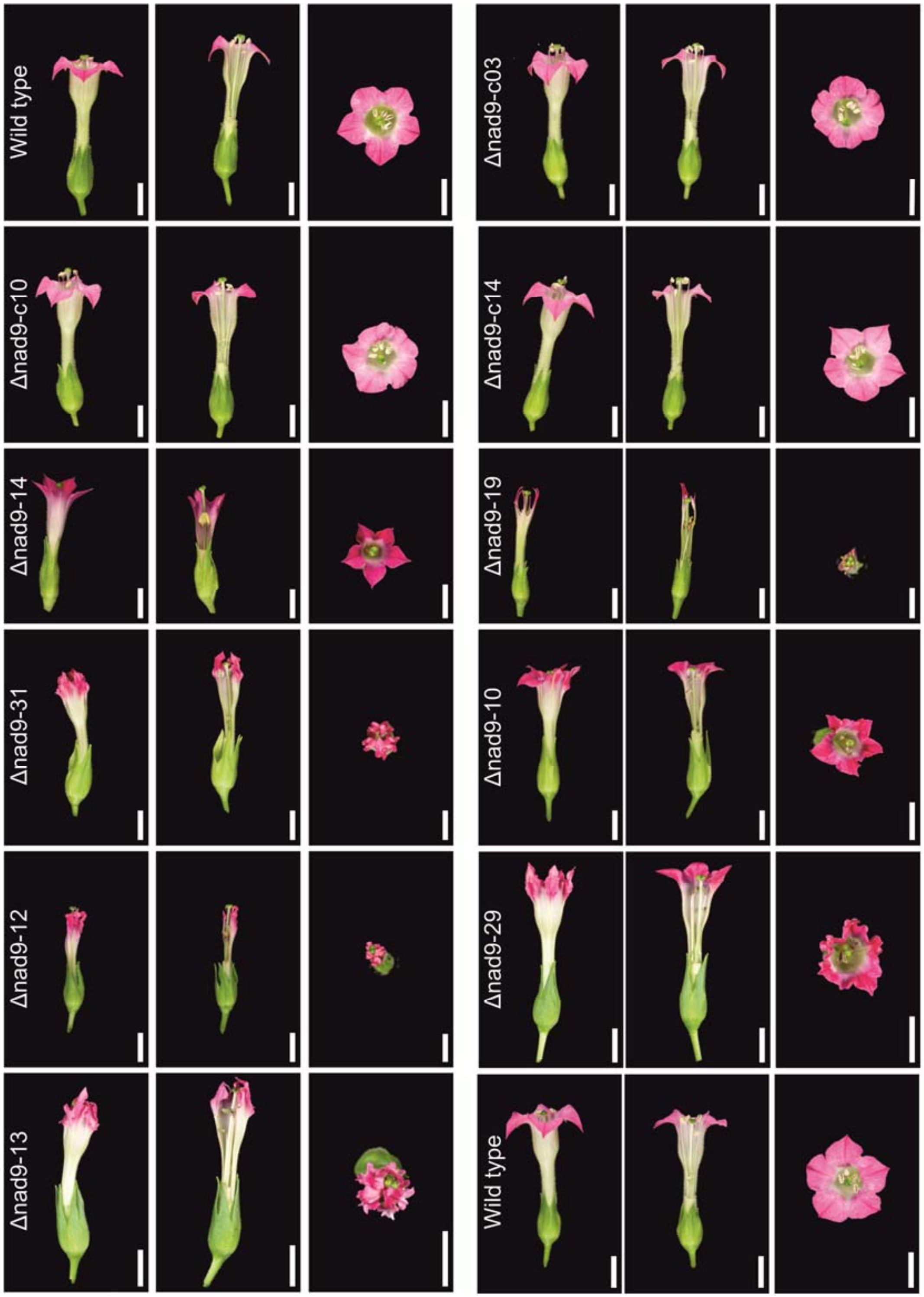

Supplementary Figure 5. Examples of flower phenotypes of wild-type plants, three independently generated allotopically complemented lines, and seven independent *nad9* deletion lines under standard greenhouse conditions. Even in an individual mutant plant,

flower phenotypes were somewhat variable, indicating limited penetrance of the aberrant morphology. Scale bars: 1 cm.

Supplementary Table 1. Recovery frequency of *nad9* deletion lines and treatments used. Rr, regeneration round; *positive in genotyping PCR; **lines successfully established; [$]23 plants were from Rr 3; [1]lines *Δnad9-05*, *Δnad9-06*, Δnad9-07, Δnad9-08, Δnad9-09, Δnad9-10, *Δnad9-11*, *Δnad9-12*, *Δnad9-13*, Δnad9-14, Δnad9-15, Δnad9-16, *Δnad9-24*, *Δnad9-25*; [2]lines Δnad9-01, *Δnad9-02*, Δnad9-03, *Δnad9-04*, *Δnad9-17*, *Δnad9-18*, Δnad9-19, Δnad9-20, Δnad9-21, Δnad9-22, Δnad9-23 (Δnad9-17 and higher from Rr 3); [3]lines Δnad9-26, Δnad9-27, Δnad9-28; [4]lines Δnad9-29, Δnad9-30, Δnad9-31; lines in magenta: ethidium bromide (EtBr) used at some point during tissue culture (19); lines in cyan: N-nitroso-N-ethylurea (NEU) used at some point during tissue culture (19); lines underlined: initial seed treated with EtBr (prior to tissue culture); lines in *italics*: initial seed treated with NEU (prior to tissue culture).

	With rotenone (mutagenesis series 1; Rr 1)	Without rotenone (mutagenesis series 1; Rr 2 and Rr 3[$])	Without rotenone (mutagenesis series 2; Rr 2)	Without rotenone (mutagenesis series 2; Rr 3)	Sum
Number of Δnad9 shoots obtained	16* (14**)[1]	12*(11**)[2]	4* (3**)[3]	5* (3**)[4]	37* (31**)
Shoots screened	20	69	60	139	288
[%] Δnad9	80 (70)	17.4 (14.5)	6.7 (5)	3.6 (2.2)	12.8 (10.8)

Supplementary Table 2. Photosynthetic parameters measured on leaves of the wild type, three *nad9* deletion lines and three allotopically complemented lines. Statistically significant differences to the wild type (One-way ANOVA, All Pairwise Multiple Comparison Procedure with Holm-Sidak method) are highlighted in red. Note that the plants measured after transfer to high light were older than those measured after growth in low light. ETRII, maximum capacity of linear electron transport; P_M, maximum amplitude of the P_{700} difference transmittance signal in the near-infrared range; F_V/F_M, maximum quantum efficiency of PSII in the dark-adapted state.

Plants grown in low light

**Parameter**	**Wild type** (n = 10)		**Δnad9-01** (n = 5)		**Δnad9-13** (n = 5)		**Δnad9-29** (n = 5)		**Δnad9-c03** (n = 10)		**Δnad9-c10** (n = 10)		**Δnad9-c14** (n = 9)	
Leaf length [cm]	**13.8**	1.8	**12.5**	0.9	**12.5**	0.9	**12.7**	0.7	**12.9**	1.6	**12.9**	1.0	**13.3**	0.8
Chlorophyll *a/b*	**3.65**	0.06	**3.59**	0.06	**3.69**	0.05	**3.57**	0.06	**3.65**	0.08	**3.68**	0.05	**3.59**	0.06
Chlorophyll content [mg m^{-2}]	**344.4**	34.1	**380.7**	44.5	**428.5**	27.5	**448.4**	16.2	**353.0**	35.7	**365.8**	11.1	**353.7**	21.0
F_V/F_M	**0.82**	0.00	**0.81**	0.01	**0.82**	0.01	**0.82**	0.01	**0.82**	0.00	**0.82**	0.00	**0.81**	0.00
ETRII [µmol m^{-2} s^{-1}]	**75.3**	8.5	**80.8**	8.1	**82.0**	9.6	**86.1**	4.5	**75.6**	7.7	**83.3**	5.1	**69.6**	4.7
P_M	**2.16**	0.28	**2.35**	0.31	**2.60**	0.21	**2.65**	0.18	**2.16**	0.25	**2.28**	0.10	**2.08**	0.15

Plants grown in high light

**Parameter**	**Wild type** (n = 10)		**Δnad9-01** (n = 9)		**Δnad9-13** (n = 10)		**Δnad9-29** (n = 10)		**Δnad9-c03** (n = 10)		**Δnad9-c10** (n = 11)		**Δnad9-c14** (n = 10)	
Leaf length [cm]	**20.0**	1.8	**19.1**	1.1	**19.0**	2.2	**20.1**	1.4	**20.6**	1.1	**20.4**	1.0	**20.3**	1.3
Chlorophyll *a/b*	**4.45**	0.20	**4.59**	0.13	**4.54**	0.31	**4.42**	0.34	**4.62**	0.21	**4.75**	0.21	**4.52**	0.13
Chlorophyll content [mg m^{-2}]	**333.0**	70.9	**639.3**	123.9	**613.2**	91.1	**651.5**	69.6	**358.3**	56.2	**323.2**	38.7	**308.2**	75.2
F_V/F_M	**0.73**	0.02	**0.79**	0.04	**0.80**	0.05	**0.82**	0.02	**0.75**	0.03	**0.75**	0.04	**0.73**	0.03
ETRII [µmol m^{-2} s^{-1}]	**124.2**	22.3	**206.9**	24.5	**178.1**	32.7	**204.1**	24.5	**136.6**	23.8	**119.3**	14.5	**121.6**	28.5
P_M	**2.72**	0.53	**4.54**	0.92	**4.02**	0.64	**4.34**	0.38	**2.96**	0.40	**2.81**	0.35	**2.48**	0.49

Supplementary Table 3. Recovered *nad9* NUMT alleles and their differences to the wild type (1117 bp). SNP, single nucleotide polymorphism; InDel, insertion or deletion; §*tomentosiformis* allele, $clone with a single additional nucleotide deviation from the mitochondrial sequence (likely reflecting an amplification error), *allele with an internal KpnI site (thus allowing recovery of the 5' part of the allele only), &clones with two additional unique nucleotide deviations each.

	**Clones**	**SNPs**	**InDels**	**Length [bp]**
Allele 01§	6	4	1	1109
Allele 02	6 + 1$	43	4	823
Allele 03	11	26	1	534*
Allele 04	9	21	0	1117
Allele 05	13	51	2	1113
Allele 06	10	27	2	1115
Allele 07	8	65	0	1117
Allele 08	10	67	0	1117
Allele 09	7	12	1	1116
Allele 10	5	65	3	1094
Allele 11	5	58	1	1117
Allele 12	7	28	1	1116
Allele 13	6 + 2&	4	0	1117
Allele 14	6	4	0	1117
Allele 15	4 + 1$	48	1	1116
Allele 16	3 + 1$	0	0	1117

Supplementary Table 4. List of oligonucleotides used in this study.

Name	Sequence (5' to 3')	Target
oAT02	AAAACAAAACATAGCTCGTGTTAACTG	*NtDHPS* (3' UTR)
oAT09	GAGTTAGCCGGTATTCCTGCTTG	*NtDHPS* (exon 2)
oJF060	GTTGTGTGGAATTGTGAGC	pGGA000
oJF061	GTTTTCCCAGTCACGACG	pGGA000
oJF271	AACAGGTACCGTTGGGAGACTTTACCCAAG	*Ntnad9* (CDS)
oJF272	TGTTGGATCCTTATCCGTCGCTACGCTGT	*Ntnad9* (CDS)
oJF273	AACAGGTACCAAGTTTCGATCGATATTTGCG	*Ntnad9* (CDS)
oJF311	CCAAGCAATGCCCAAAAGTCCCATGC	*Ntnad9* (5' UTR)
oJF401	CAGATAGCTGGGCAATGGAATCC	*35S* promoter
oJF469	AACAGGATCCGCACCAGCTGGGCCTCTCTCTT	*Ntnad9* (upstream sequence)
oJF470	TGTTGGTACCCTCAATGATATCCGACGATCAAGAC	*Ntnad9* (downstream sequence)
oJF481	CCCAAATGAAGTGCAGGTCA	*ocs* terminator
oJF496	GAGTTGATTATCCCTCTCGA	*Ntnad9* (CDS)
oJF499	TCTTGGGTCATCTCAATGGG	*Ntnad9* (CDS)
oJF740	AACAGGTCTCAACCTCGACGAGTCAGTAATAAACGG	*AtUBQ10* promoter
oJF748	ATGCCGGACATCTTAAGGAG	adapter sequence (for cDNA synthesis)
oJF822	TGTTGGTCTCTTGTTCTGTTAATCAGAAAAACTCAGATTAATCTAC	*AtUBQ10* promoter
oJF883	GCTAAAGGTAGCTTGTCTCG	*Ntrrn18*
oJF884	CACTCATCGTTTACGGCATG	*Ntrrn18*
oJF937	TCAAGGTACCAACAATGGGTAAAAAGCCTGAACTC	*hpt* CDS (hygromycin resistance)
oJF938	TGACGAGCTCCTATTCCTTTGCCCTCGGACGA	*hpt* CDS (hygromycin resistance)
oJF939	ATAGGAGCTCGTCAAGCAGATCGTTCAAACATTTGGC	*nos* terminator
oJF940	TGTTGGTACCTTGAGAGTGAATATGAGACTC	*nos* promoter
oJF947	AGTGAAGCTTGGTCTCAAACAATGGC	multiple cloning site of pJF1270
oJF948	GCGAGAATTCGGTCTCAGCAGT	multiple cloning site of pJF1270
oJF1026	ATGCCGGACATCTTAAGGAGTCTGTACTGCTGAGAACCAC	*β-TUBULIN* (CDS)
oJF1028	GAACCTTACAATGCTACTCTGTC	*β-TUBULIN* (CDS)
oJF1029	TCTGTACTGCTGAGAACCAC	*β-TUBULIN* (CDS)
oJF1088	ATGCCGGACATCTTAAGGAGAAACGCTGCTTCCTAGATGG	*NtNAD9* (CDS)

oJF1090	CTTGTGTTCACTCGTGAGCT	*StFDH* (CDS)
oJF1113	ATGCCGGACATCTTAAGGAGAATAGCATTTCCTATTGATTTGTCC	*Ntnad9* (3' UTR)
oJF1144	ATGCCGGACATCTTAAGGAGAAATGGGACCGCGAAGACAAC	*Ntatp9* (3' UTR)
oJF1145	CCTATGCTTTGCATGAACATCTC	*Ntatp9* (5' UTR)
oJF1147	GTGTATCCAAGTTTGCGCAAG	*AtIVD* (CDS)
oJF1323	AGCGACGGTCCCTAACGCT	TALEN (CDS)
oJF1369	TGATCGTAGATAGAGACTTCAACC	*NttrnH-GUG* (upstream sequence)
oJF1374	AATGGATCCCTCGTTACTGC	*NttrnP-UGG* (downstream sequence)
