# Supplementary material for: Targeted knockout of a conserved plant mitochondrial gene by genome editing
Source: Nat Plants. 2023 Oct 9;9(11):1818–31. doi: 10.1038/s41477-023-01538-2 (PMC10654050; doi:10.1038/s41477-023-01538-2)
Supplement: Supplementary file 2 — Reporting Summary [file 41477_2023_1538_MOESM2_ESM.pdf]

nature portfolio

Corresponding author(s): Ralph Bock

Last updated by author(s): Sep 1, 2023

Reporting Summary

Nature Portfolio wishes to improve the reproducibility of the work that we publish. This form provides structure for consistency and transparency in reporting. For further information on Nature Portfolio policies, see our Editorial Policies and the Editorial Policy Checklist.

Statistics

For all statistical analyses, confirm that the following items are present in the figure legend, table legend, main text, or Methods section.

n/a	Confirmed	
☐	☒	The exact sample size (*n*) for each experimental group/condition, given as a discrete number and unit of measurement
☒	☐	A statement on whether measurements were taken from distinct samples or whether the same sample was measured repeatedly
☐	☒	The statistical test(s) used AND whether they are one- or two-sided<br>*Only common tests should be described solely by name; describe more complex techniques in the Methods section.*
☒	☐	A description of all covariates tested
☒	☐	A description of any assumptions or corrections, such as tests of normality and adjustment for multiple comparisons
☐	☒	A full description of the statistical parameters including central tendency (e.g. means) or other basic estimates (e.g. regression coefficient) AND variation (e.g. standard deviation) or associated estimates of uncertainty (e.g. confidence intervals)
☒	☐	For null hypothesis testing, the test statistic (e.g. *F*, *t*, *r*) with confidence intervals, effect sizes, degrees of freedom and *P* value noted<br>*Give P values as exact values whenever suitable.*
☒	☐	For Bayesian analysis, information on the choice of priors and Markov chain Monte Carlo settings
☒	☐	For hierarchical and complex designs, identification of the appropriate level for tests and full reporting of outcomes
☒	☐	Estimates of effect sizes (e.g. Cohen's *d*, Pearson's *r*), indicating how they were calculated

Our web collection on statistics for biologists contains articles on many of the points above.

Software and code

Policy information about availability of computer code

Data collection	Sequencing was carried out in an Illumina MiSeq micro flow cell in PE150bp mode yielding between 220,000 and 300,000 fragments per sample.
Data analysis	The initial quality check of the sequence data of all samples (in FASTQ format) was done with FastQC v. 0.11.9 9 (https://www.bioinformatics.babraham.ac.uk/34projects/fastqc/). Clipping of adapters was performed with CLCGenomicsWorkbench v. 22 (https://digitalinsights.qiagen.com/products-overview/discovery-insightsportfolio/analysis-and-visualization/qiagen-clc-genomics-workbench/). The trimmed FASTQC files of all datasets were then mapped with bwa v. 0.7.17 in mem mode (https://doi.org/10.48550/arXiv.1303.3997) against the NCBI NC_006581.1 (https://www.ncbi.nlm.nih.gov/nuccore/NC_006581) reference followed by postprocessing using SAMTools v. 1.14 (57; http://www.htslib.org/). Scanning for structural variants was done by DELLY v. 1.0.3 (58; https://github.com/dellytools/delly/), after marking duplicates with picard v. 2.27.5 (https://github.com/broadinstitute/picard/releases/tag/2.27.5) and IGV v. 2.13.2 (59; https://software.broadinstitute.org/software/igv/) and by using the mpileup feature of SAMTools. De novo assembly of sample Δnad9-29 was done manually by removing the deleted sequence from the wild type FASTA file (NC_006581.1). The annotation of the assembled sequences was conducted with the GeSeq tool v. 2.03 (60), and OGDRAW v. 1.3.1 was used for graphical representation of the genomes (61, 62; https://chlorobox.mpimpgolm.mpg.de/).<br>DNA sequence analyses were performed using the tools of the Lasergene suite (DNASTAR) versions 14 and 17, the SnapGene Viewer v. 7.0.1 (https://www.snapgene.com/snapgene-viewer/), and BLAST (https://blast.ncbi.nlm.nih.gov).<br>Images were analyzed using Adobe Photoshop CS5 Extended v. 12.0.4 and Fiji (ImageJ 1.52i).

For manuscripts utilizing custom algorithms or software that are central to the research but not yet described in published literature, software must be made available to editors and reviewers. We strongly encourage code deposition in a community repository (e.g. GitHub). See the Nature Portfolio guidelines for submitting code & software for further information.

Data

Policy information about availability of data

All manuscripts must include a data availability statement. This statement should provide the following information, where applicable:

- Accession codes, unique identifiers, or web links for publicly available datasets
- A description of any restrictions on data availability
- For clinical datasets or third party data, please ensure that the statement adheres to our policy

The data supporting the findings of this study are available within the paper and its supplementary information files. The full sequence of pJF1271 has been deposited in GenBank under accession number OQ418153 (https://www.ncbi.nlm.nih.gov/nuccore/OQ418153). The NGS sequencing results are available under https://www.ncbi.nlm.nih.gov/bioproject/?term=PRJNA934725 as stated in the manuscript. Access is not restricted. NCBI entry NC_006581.1 (https://www.ncbi.nlm.nih.gov/nuccore/NC_006581) was used as the mitochondrial reference genome for tobacco.

Human research participants

Policy information about studies involving human research participants and Sex and Gender in Research.

Reporting on sex and gender	not applicable
Population characteristics	not applicable
Recruitment	not applicable
Ethics oversight	not applicable

Note that full information on the approval of the study protocol must also be provided in the manuscript.

Field-specific reporting

Please select the one below that is the best fit for your research. If you are not sure, read the appropriate sections before making your selection.

☒ Life sciences ☐ Behavioural & social sciences ☐ Ecological, evolutionary & environmental sciences

For a reference copy of the document with all sections, see nature.com/documents/nr-reporting-summary-flat.pdf

Life sciences study design

All studies must disclose on these points even when the disclosure is negative.

Sample size	No sample size calculation was performed as we had no prior knowledge about the efficiency of our method. We stopped after obtaining 31 positive lines, because this clearly demonstrates feasibility of the method.
Data exclusions	No data was excluded.
Replication	Work was carried out repeatedly (more than 3 attempts) for line establishment. Line characterization was done with several (3 to 31) independent lines in parallel. As nad9 deletion lines were obtained and confirmed in all attempts, all attempts at replication were successful.
Randomization	Randomization was not relevant to the study. The study focuses on demonstrating feasibility, not on comparing different groups/treatment.
Blinding	Blinding was not relevant to the study. The study focuses on demonstrating feasibility, not on comparing different groups/treatment.

Reporting for specific materials, systems and methods

We require information from authors about some types of materials, experimental systems and methods used in many studies. Here, indicate whether each material, system or method listed is relevant to your study. If you are not sure if a list item applies to your research, read the appropriate section before selecting a response.

Materials & experimental systems

n/a	Involved in the study
☐	☒ Antibodies
☒	☐ Eukaryotic cell lines
☒	☐ Palaeontology and archaeology
☒	☐ Animals and other organisms
☒	☐ Clinical data
☒	☐ Dual use research of concern

Methods

n/a	Involved in the study
☒	☐ ChIP-seq
☒	☐ Flow cytometry
☒	☐ MRI-based neuroimaging

Antibodies

Antibodies used	Primary antibodies: anti-Nad9 (reference 54) and anti-Cox1 (reference 55).<br>Secondary antibody: anti-rabbit-HRP conjugate from Sigma (A0545).
Validation	For validation of primary antibodies, see references 54 & 55; for secondary antibody see https://www.sigmaaldrich.com/DE/en/product/sigma/a0545.
