# Supplementary figures and images for: Targeted knockout of a conserved plant mitochondrial gene by genome editing
Source: Nat Plants. 2023 Oct 9;9(11):1818–31. doi: 10.1038/s41477-023-01538-2 (PMC10654050; doi:10.1038/s41477-023-01538-2)

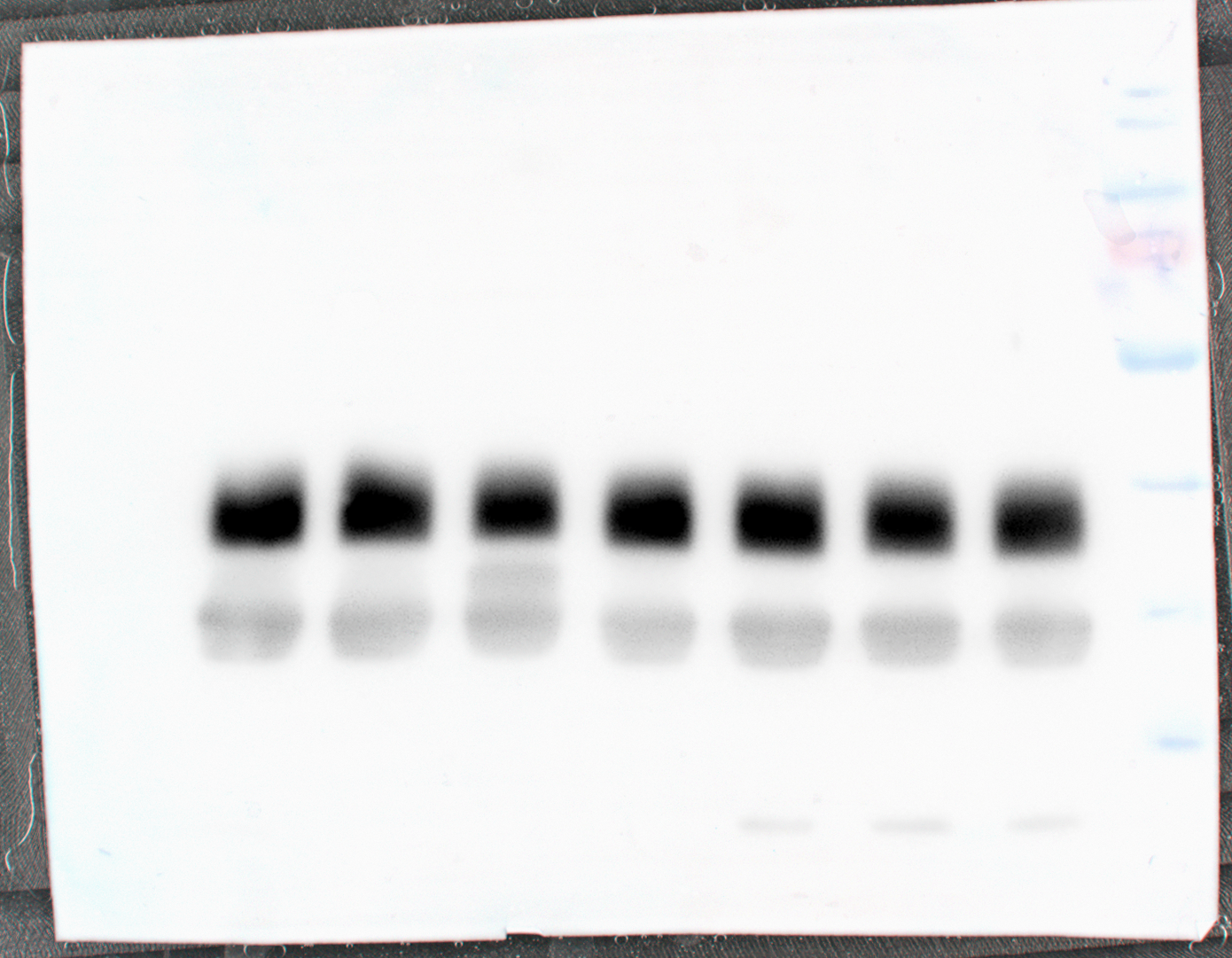

Supplement: Supplementary file 3 — Unprocessed western blot for Fig. 5b (lowest panel). [file 41477_2023_1538_MOESM3_ESM.tif]
